# Draft genome sequence of bitter gourd (*Momordica charantia*), a vegetable and medicinal plant in tropical and subtropical regions

**DOI:** 10.1093/dnares/dsw047

**Published:** 2016-12-17

**Authors:** Naoya Urasaki, Hiroki Takagi, Satoshi Natsume, Aiko Uemura, Naoki Taniai, Norimichi Miyagi, Mai Fukushima, Shouta Suzuki, Kazuhiko Tarora, Moritoshi Tamaki, Moriaki Sakamoto, Ryohei Terauchi, Hideo Matsumura

**Affiliations:** 1 Okinawa Prefectural Agricultural Research Center, Itoman, Okinawa 901-0336, Japan; 2 Iwate Biotechnology Research Center, Kitakami, Iwate 024-0003, Japan; 3 Shinshu university, Ueda, Nagano 386-8567, Japan

**Keywords:** Cucurbitaceae, *Momordica charantia*, bitter gourd, draft genome, *de novo* sequencing

## Abstract

Bitter gourd (*Momordica charantia*) is an important vegetable and medicinal plant in tropical and subtropical regions globally. In this study, the draft genome sequence of a monoecious bitter gourd inbred line, OHB3-1, was analyzed. Through Illumina sequencing and *de novo* assembly, scaffolds of 285.5 Mb in length were generated, corresponding to ∼84% of the estimated genome size of bitter gourd (339 Mb). In this draft genome sequence, 45,859 protein-coding gene loci were identified, and transposable elements accounted for 15.3% of the whole genome. According to synteny mapping and phylogenetic analysis of conserved genes, bitter gourd was more related to watermelon (*Citrullus lanatus*) than to cucumber (*Cucumis sativus*) or melon (*C. melo*). Using RAD-seq analysis, 1507 marker loci were genotyped in an F_2_ progeny of two bitter gourd lines, resulting in an improved linkage map, comprising 11 linkage groups. By anchoring RAD tag markers, 255 scaffolds were assigned to the linkage map. Comparative analysis of genome sequences and predicted genes determined that putative trypsin-inhibitor and ribosome-inactivating genes were distinctive in the bitter gourd genome. These genes could characterize the bitter gourd as a medicinal plant.

## 1. Introduction

Bitter gourd (*Momordica charantia*, 2*n* = 2*x* = 22^1^) is a dicot vine species belonging to the family Cucurbitaceae originating in tropical Asia. Bitter gourd, also known as African cucumber, bitter cucumber, bitter melon, balsam pear, or karela in the region,^2^^,^^3^ is characterized by its warty-skinned fruit and is widely cultivated in tropical and subtropical regions of the world. The flesh of bitter gourd fruit tastes bitter owing to the presence of the cucurbitacin-like alkaloid, momordicine, and triterpene glycosides. Bitter gourd fruit are rich in vitamin C and phenolic compounds with antioxidant activity.^4–7^ Additionally, leaf decoction of bitter gourd is used in traditional medicine for the treatment of stomach pain, anemia, malaria, coughs, and fever.^8^ Recently, several studies have shown its antidiabetic effect *in vitro* and *in vivo*.^9–11^ Therefore, these properties have given the plant a high medicinal value and made it the subject of recent scientific research. Similar to other Cucurbitaceae crops, bitter gourd is a monoecious plant species. However, some gynoecious lines have been found,^4^ providing useful genetic resources (as maternal plants) in breeding programs for the production of F_1_ hybrids. Matsumura *et al.*^12^ succeeded in genetically mapping the locus responsible for gynoecy and identified restriction-associated DNA tag sequencing (RAD-seq) markers linked to the locus. In *Cucumis* spp., sex determination has been well studied, and ethylene has been shown to play a key role in its regulation. Genes encoding aminocyclopropane-1-carboxylic acid (ACC) synthase have been shown to be responsible for gynoecy, unisexual flower development, and andromonoecy in cucumber or melon.^13–15^ Through silver nitrate mediated inhibition of ethylene, production of female flowers in the gynoecious bitter gourd was obstructed in favor of bisexual flowers, indicating the possible involvement of ethylene in the sex determination. However, because of the limited genome sequence information in comparison to cucumber,^16^ melon,^17^ and watermelon,^18^ the genes underlying sex determination in bitter gourd are yet to be identified.

In this study, we determined the whole genome sequence of bitter gourd, which was generated through the Illumina next-generation sequencing platform followed by *de novo* assembly. *Ab initio* gene prediction and annotation of predicted genes were also carried out. Based on these assembled genome sequences and gene prediction, the bitter gourd genome was compared with known genome sequences of other Cucurbitaceae species. Additionally, through RAD-seq analysis, a linkage map was constructed onto which the assembled scaffolds were assigned. These results provided a basis for gene identification and DNA marker development in bitter gourd, and a platform for studying evolution in Cucurbitaceae species.

## 2. Materials and methods

### 2.1. Plant materials and DNA preparation

A monoecious inbred line OHB3-1 developed by the Okinawa Prefectural Agricultural Research Center was used for *de novo* sequencing of the bitter gourd genome. Genomic DNA was extracted from young leaves using a NucleoSpin Plant II kit (Macherey-Nagel) according to the manufacturer’s instructions. For RAD-seq analysis, two parental bitter gourd lines, OHB61-5 and OHB95-1A, and their F_2_ progeny^12^ were used as materials.

### 2.2. Library preparation and sequencing

Sequencing libraries were prepared from genomic DNA for Illumina MiSeq and HiSeq2500 platforms. A short insert (330 bp) paired-end (PE) library was constructed using a TruSeq DNA PCR-Free LT Sample Prep Kit (Illumina), which reduced PCR amplification bias in library preparation. Mate-paired (MP) libraries with various insert sizes (2, 4, 6, and 8 kbp) were constructed using the Nextera Mate Pair Sample Prep Kit (Illumina). The PE library was sequenced using MiSeq (2 × 230 bp) and the four MP libraries were sequenced using HiSeq2500 (2 × 100 bp).

### 2.3. Sequence assembly

Sequence reads in fastq files from MiSeq and HiSeq2500 were quality-filtered by FASTX-Toolkit version 0.0.13 (http://hannonlab.cshl.edu/fastx_toolkit/). For *de novo* assembly, sequence reads with a PHRED quality score of ≥ 30 comprising of ≥ 90% of the reads were extracted. After adaptor trimming and removal of reads with inappropriate insert sizes in MP libraries using an in-house pipeline of scripts, qualified reads (Supplementary Table S3) were applied to *de novo* assembly using ALLPATHS-LG assembler version R49856^19^ with setting PLOIDY = 2 and HAPLOIDIFY = True. All the constructed scaffold sequences were aligned with each other using BLASTN, and perfectly identical scaffolds to others in entire sequences, were excluded as duplicated scaffolds. In the remaining scaffolds showing similarity to others, when the observed frequency of mismatch and indel sites per 1,000 bases was less than 1 in both aligned scaffold sequences, they were presumed to be allelic.

For mitochondrial (Mt) or chloroplast (Cp) genome sequences, PE and MP reads were aligned to 80 and 697 Mt and Cp reference genome sequences in the NCBI database (Supplementary Tables S1 and S2) using Burrows-Wheeler Aligner (BWA) version 0.6.1 with default parameters, respectively. Mapped reads to Mt or Cp reference sequences were extracted from the original fastq files, and applied to the assembly using ALLPATHS-LG as described above.

### 2.4. Gene prediction and annotation

Gene prediction analysis in the bitter gourd scaffold sequences was carried out using *ab initio* prediction by FGENESH software ver 3.1.1 (Softberry)^20^ based on Hidden Markov Model (HMM)-based gene prediction (Supplementary method). For annotating predicted genes, encoded protein sequences were applied to the BLASTP search against the non-redundant (NR) protein database in NCBI (ftp://ftp.ncbi.nlm.nih.gov/blast/db/) and UniProtKB/Swiss-Prot database (http://www.uniprot.org), respectively. As domain searches of encoded proteins of predicted genes, all the amino acid sequences were applied to InterProscan version 5.19-58.0 (https://www.ebi.ac.uk/interpro/) with default settings (Supplementary method). Transposable elements in the predicted genes were identified using TransposonPSI (http://transposonpsi.sourceforge.net/), and only the top hits against individual library searches with default settings were employed for their annotation.

### 2.5. Comparative analysis of genomes among Cucurbitaceae species

Comparison of the bitter gourd genome and other Cucurbitaceae genomes was performed by mapping OHB3-1 scaffold sequences to cucumber (cucumber_ChineseLong_v2_genome, http://www.icugi.org/cgi-bin/ICuGI/index.cgi),^16^ melon (CM3.5.1_pseudomol, https://melonomics.net/),^17^ and watermelon (WCG_chromosome_v1, http://www.icugi.org/cgi-bin/ICuGI/index.cgi)^18^ genome sequences using SyMap 4.2 (http://www.agcol.arizona.edu/software/symap/).^21^

### 2.6. Conserved genes among Cucurbitaceae species and unique genes in bitter gourd genome

By comparing a list of anchors analyzed by the SyMap program, genes showing conserved synteny among all four Cucurbitaceae species were identified, and applied to phylogenetic analysis using Aminosan^22^ and RAxML^23^ as described in Supplementary method.

To identify genes showing unique structures in the bitter gourd genome, anchor gene lists in the SyMap analysis were compared in all four Cucurbitaceae species. Unanchored genes to any predicted genes in melon, cucumber, or watermelon genomes, were found. Reversely, conserved (syntenic) genes among melon, cucumber, and watermelon genomes, but not in the bitter gourd genome, were also found. Functional annotation of these selected genes was determined by domain searches of encoded protein sequences using InterProScan as described above.

### 2.7. RAD-seq analysis

RAD-seq analysis was performed as described previously.^12^ Briefly, genomic DNA was digested with *Ase*I restriction endonuclease, and a biotinylated adapter, harboring index sequences, was ligated to the digested DNA fragments. The adapter ligated genomic DNA fragments were then digested with *Nla*III restriction endonuclease. Biotinylated fragments were collected using streptavidin-coated magnetic beads (Dynabeads M270, Thermofisher), and the additional adapter was ligated to the end of the fragments on the magnetic beads. These adapter-ligated fragments on the beads were amplified by PCR. The PCR products were then sequenced using the HiSeq2500 system. From the sequence reads, 80 bp sequences including *Ase*I-recognition sites were extracted as RAD-seq tags. Tag extraction and counting was carried out using CLC Genomics Workbench software (Qiagen).

### 2.8. Reference mapping of RAD-seq tags

Tag sequences showing more than 20 counts in either parent line (OHB61-5 or OHB95-1A) were employed in further analysis. These tag sequences (80 bp) were mapped to the scaffold sequences of OHB3-1 as ‘reference sequences’ using BWA version 0.6.1 in DDBJ Read Annotation Pipeline (https://p.ddbj.nig.ac.jp/pipeline/). Procedures for detection of polymorphic or heterozygous loci were described in Supplementary method.

### 2.9. Linkage map development

An RAD-seq analysis of 97 F_2_ plants derived from OHB61-5 and OHB95-1A was carried out as described above. Based on analyzed RAD-seq data in individual F_2_ plants, genotypes of bi-allelic tags as co-dominant markers were determined following a previously described method.^12^ Genotyping procedure and a linkage map construction using JoinMap4.1 (Kyazma)^24^ were described in Supplmentary method.

### 2.10. Comparative analysis of orthologous and paralogous genes

Homologues of genes for putative trypsin inhibitor, ribosome inactivating protein, ACC synthase and CmWip1 were identified by BLAST searches against predicted genes in melon, cucumber and watermelon genome. Sequence alignment and phylogenetic analysis was performed using MEGA7.0.18.^25^ Detail of analysis was described in Supplementary method.

### 2.11. RT-PCR analysis

Total RNA was extracted from flower buds of bitter gourd plant, and expression of sex determination-related genes was analyzed by RT-PCR as described in Supplementary method.

## 3. Results and discussion

### 3.1. Sequencing and assembly of the bitter gourd genome

In the current study, a monoecious inbred line (OHB3-1) of bitter gourd was used for genome sequencing using the Illumina platform. Paired-end (PE) and mate-pair (MP; with 2, 4, 6, and 8 kbp inserts) libraries were constructed from genomic DNA and sequenced using the Illumina MiSeq or HiSeq2500 DNA sequencer. For PE library development, PCR amplification was avoided and long read sequencing (2×230 bp) was carried out. The total length of the analyzed sequence reads amounted to over 37 Gb (Supplementary Table S3), which was equivalent to approximately 110 times that of the estimated genome size (339 Mb) of bitter gourd,^26^ representing a sufficient quantity of sequence reads for whole genome assembly. Using these sequence reads, scaffolds were constructed using the ALLPATHS-LG assembler.^19^ Using BLAST analysis of the assembled scaffolds of each other, six pairs of putative allelic scaffolds (scaffold_617 and 614, 950, and 911, 988 and 901, 699 and 700, 690 and 691, 657 and 604), which contained mismatch and/or indel sites, were found. However, since it was difficult to discriminate between allelic and paralogous sequences in this study, they were included in the draft genome sequence data as independent scaffolds in this study. The total length of the assembled scaffolds was 285.5 Mb, which comprised 1,029 scaffolds (Table 1), corresponding to approximately 84% of the previously estimated genome size.^26^ The N50 value of these scaffolds was 1.1 Mb, and the longest scaffold sized was over 7 Mb (Table 1). According to previous studies, coverage (%) and N50 values of assembled sequences were 66% and 1.1 Mb in cucumber,^16^ 83% and 4.7 Mb in melon,^17^ and 83% and 2.3 Mb in watermelon,^18^ respectively. The present genome assembly of bitter gourd is comparable to the assembly of other cucurbits genomes. However, 15% of the genome was undetermined in the present sequencing analysis. It is possible that the redundant regions of the genome, such as sequences encoding multiple copies of repeats or transposons, interfered with accurate assembly, resulting in shorter assembled scaffolds than the actual complete genome. Among the scaffolds of the OHB3-1 genome, sequences of 94,148 ambiguous degenerate bases (0.03%) were present, possibly owing to heterozygous loci or assembly of redundant regions (data not shown).

**Table 1.** Summary of assembly results in OHB3-1 genome sequence

Nuclear genome	
Scaffold number	1,029
Total length (bp)	285,543,823
N50 (bp)	1,100,631
Maximum length (bp)	7,185,522
GC content (%)	36.4
Mitochondrial genome	
Scaffold number	1
Total length (bp)	312,781
GC content (%)	41.1
Chloroplast genome	
Scaffold number	3
Total length (bp)	140,659
Maximum length (bp)	131,815
GC content (%)	35.8

Based on 80 mitochondrial and 697 chloroplast reference genome sequences (Supplementary Tables S1 and S2), scaffolds for organelle genome were developed. Scaffold length of the mitochondrial genome was 312,781 bp, and the total length of three scaffolds of the chloroplast genome was 40,659 bp (Table 1). Through the BLAST search, the scaffold sequence of the bitter gourd mitochondrial genome showed high similarity to the watermelon mitochondrial genome sequence (Supplementary Table S4). In the assembled chloroplast genome, scaffold1, scaffold2, and scaffold3 showed the highest similarity to the plastid or chloroplast genome of five-leaf ginseng (*Gynostemma pentaphyllum*) or cucumber, bottle gourd (*Lagenaria siceraria*), and melon, respectively (Supplementary Table S4).

### 3.2. Gene prediction and transposon exploration

Genes in the scaffold sequence of OHB3-1 were inferred by an *ab initio* prediction using the FGENESH program.^20^ In total, 45,859 protein-coding genes were found as predicted genes in the OHB3-1 scaffold sequence (Table 2, Supplementary Table S5). Their average number of CDS (coding sequences) per a predicted gene was 4.41 and 8,512 genes constituted only a single CDS. Length of encoded protein in these predicted genes was 331 a.a on average. Transcription start sites and polyadenylation sites in the predicted genes were also found in 45,267 and 45,799 genes, respectively. Gene content in the bitter gourd scaffolds was more than that in the other sequenced Cucurbitaceae genomes (26,682 in cucumber,^16^ 27,427 in melon,^17^ and 23,440 in watermelon^18^). This is possibly because transcript information, such as EST (expressed sequence tag) data, was also incorporated in the gene prediction in other Cucurbitaceae genomes. Annotation of predicted genes was performed by a BLASTP search of their encoded protein sequences against non-redundant (NR) protein and UniProt database (Supplementary Table S6). Consequently, encoded proteins of 34,986 and 25,348 predicted genes showed a similarity to the sequences in NR and UniProt databases, respectively. Most of them (25,268 proteins) showed hits to both the NR and UniProt databases, whereas 80 proteins showed only hits to sequences in the UniProt, but not the NR database. In these predicted genes, 8,839 genes encoded putative transposons as determined through TransposonPSI analysis (Supplementary Table S7, Table 2). Sequences of these putative transposons (43,834 kb) accounted for ∼15.3% of the total scaffolds of the OHB3-1 genome. The majority (65%) of them belonged to the long terminal repeat (LTR) retrotransposons, Ty1/copia or Ty3/gypsy. The Ty3/gypsy type was the most abundant (35.6%), covering 5.5% of the total genome. Considering class II transposons (DNA transposons), the CACTA family was the most abundant (24.7%), comprising 3.8% of the total scaffold. For annotating predicted genes, domain searching was also carried out by InterProscan. Domain search results of their encoded protein sequences against Pfam, SMART, ProDom, and PRINTS databases were indicated in Supplementary Table S8. In total, putative encoded proteins for 24,183 genes had any conserved domains (Table 2). Of the unannotated genes by BLAST, conserved domains were found in 23 predicted genes. Consequently, BLAST and conserved domain searching resulted in the annotation of 36,086 predicted genes (∼75% of the predicted genes) in total.

**Table 2.** Summary of predicted genes in bitter gourd (OHB3-1) scaffold sequence

Total predicted genes^a^	Average length (aa) of encoded protein^a^	Annotated genes^b^			Putative transposable elements^c^
		BLAST (NR)	BLAST (UniProt)	InteProScan	
45,859	358	34,986	25,348	24,183	8,839

^a^Prediction of protein-coding genes and their translated sequences were conducted by FGENESH.

^b^Encoded amino acid sequences of the predicted genes were applied to BLASTP searching against non-redundant protein database in NCBI and UniProtKB/Swiss-Prot database or InterProscan analysis for conserved domain searching.

^c^Transposable elements in the predicted genes were surveyed by TranposonPSI.

### 3.3. Similarity of genome sequences in bitter gourd with Cucurbitaceae species

A comparison of the bitter gourd genome with those of other Cucurbitaceae crops was performed by synteny mapping of the OHB3-1 scaffolds (285.5 Mb) against pseudomolecule sequences of cucumber, melon and watermelon using the SyMap 4.2 program.^21^ In this analysis, genome or scaffold sequences of two species were aligned and ‘anchors’, which allowed the connection of two genomes, were determined by filtering based on annotated gene (predicted gene) information. Synteny blocks were defined as regions consisting of more than seven anchors between two species. Synteny blocks against the bitter gourd scaffolds covered 80–90% of each cucurbit genome sequence (Supplementary Table S9). In the watermelon genome, a few synteny blocks of a long length (>10 Mb) were observed, whereas only short (<1 Mb) fragmented blocks were mapped in the melon and cucumber genomes (Supplementary Table S9, Supplementary Figs S1–S3), implying relative structural similarities between the bitter gourd and watermelon. In the SyMap analysis, conserved genes connected between the bitter gourd scaffold and other cucurbit genomes were identified as anchors. In the bitter gourd predicted genes, 16,820, 16,063, and 16,083 genes were defined as anchors (Supplementary Table S10), which corresponded to genes in the watermelon, melon, and cucumber genome, respectively, and 14,775 loci presumed to be conserved in all compared Cucurbitaceae species. Of the genes at these loci, multiplicated (redundant) genes in each genome or scaffold sequences were eliminated, and 69 loci were defined as unique in each cucurbit genome and conserved among all four species (Supplementary Table S11). Based on the alignment of encoded amino acid sequences of these orthologous genes at each locus (Supplementary Table S12), phylogenetic relationships were analyzed by RAxML as described in Supplementary method. According to the constructed phylogenetic tree (Supplementary Fig. S4), bitter gourd was related to watermelon, rather than *Cucumis* spp., but it was evolutionary distant from other species. Previous studies suggested bitter gourd was more closely related to watermelon than to cucumber or melon, according to the internal transcribed spacer regions of nuclear ribosomal RNA genes^27^ or sequences of chloroplast genes.^28^ Our results of synteny mapping and phylogenetic analysis seemed to support these results, but further information of genome sequences in more Cucurbitaceae species is necessary to elucidate their phylogenetic relationships precisely.

### 3.4. Unique gene finding in the bitter gourd genome

**Figure 1:**
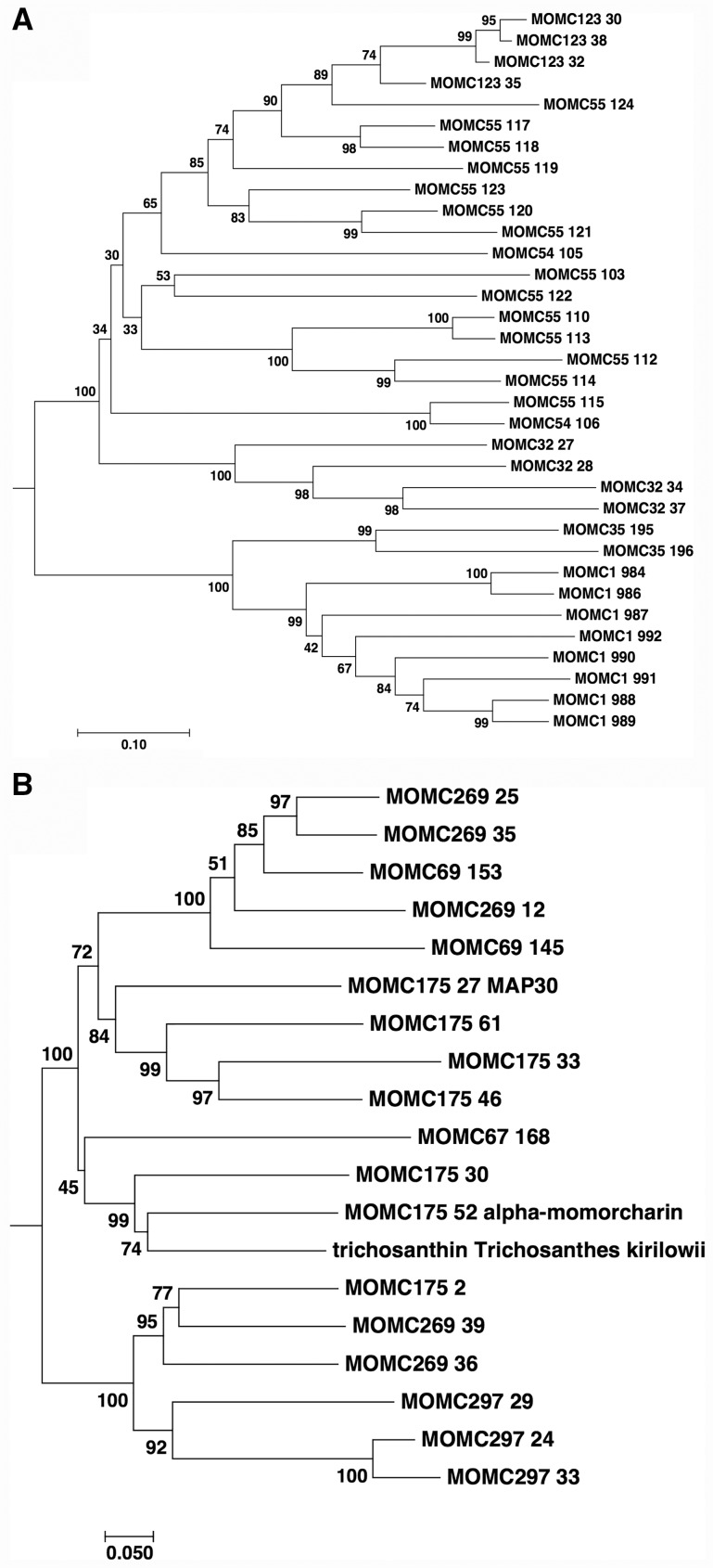
Phylogenic analysis of putative trypsin inhibitor (A) and ribosome inactivating protein (B) in bitter gourd. Based on 34 amino acid sequences of trypsin inhibitor proteins and 18 amino acid sequences of ribosome inactivating proteins of bitter gourd (Supplementary Table S12, S14, and S15), phylogenetic tree for each protein family was constructed using the Neighbor-Joining method by MEGA7.0.18. The percentage of replicate trees in which the associated taxa clustered together in the bootstrap test (500 replicates) are shown next to the branches. The tree is drawn to scale, with branch lengths in the same units as those of the evolutionary distances used to infer the phylogenetic tree. The evolutionary distances were computed using the p-distance method and are in the units of the number of amino acid differences per site.

The synteny mapping analysis by using SyMap allowed to identify unique genes and gene orders in bitter gourd scaffolds. By comparing anchor gene lists (Supplementary Table S10), 3,158 annotated genes in the bitter gourd scaffolds did not correspond to any genes in other cucurbits genomes. Reversely, 2,468 genes were conserved in the genome of three cucurbit species but absent in the bitter gourd scaffolds. Comparing the functional annotation of these characteristic genes (uniquely present or absent) in bitter gourd genome, two gene classes were distinguished. Predicted genes encoding putative trypsin inhibitor-like proteins were more frequently observed in the bitter gourd genome than other cucurbits genomes (Supplementary Table S13), and 29 genes encoding trypsin inhibitor-like proteins were clustered in the non-syntenic scaffold regions to the other three Cucurbitaceae genomes (Supplementary Table S14). No known conserved domain was found in encoded proteins of 10 genes (Supplementary Table S14), but showed a sequence similarity to cucumber trypsin inhibitor-like proteins by the BLAST search. Of these genes, three genes in scaffold_32 (*MOMC32_28g*, *MOMC32_34g*, and *MOMC32_37g*) encoded identical or homologous proteins to Mch-1 or Mch-2,^29^ which were isolated from bitter gourd seeds. Although the conserved domain as the trypsin inhibitor was not observed in these proteins, it was shown that they had trypsin inhibition activity.^29^ In another study, mcIRBP, corresponding to MOMC1_984, was isolated from bitter gourd seeds as an insulin receptor-binding protein, and its injection reduced blood glucose levels in mice,^30^ implying its possible application to anti-diabetic medication. Phylogenetic analysis of putative trypsin inhibitors in bitter gourd showed that proteins with the I7 protease inhibitor and I13 protease inhibitor domains were separated, and several proteins without conserved domains (Supplementary Table S14) were categorized in the I7 protease inhibitor group. MOMC32_27, MOMC32_28, MOMC32_34 (Mch-2), and MOMC32_37 (Mch-1) formed a monophyletic group, close to I7 protease inhibitors (Fig. 1).

Additional notably unique genes in bitter gourd genome were ribosomal inactivating protein (RIP) genes. RIP is known as a plant toxin, which has N-glycosidase activity against adenine nucleotide in ribosomal RNA.^31^ Most RIPs were classified into two groups (type 1 and type 2).^31^ Type 1 RIP is a monomeric protein encoding the N-glycosidase activity domain (A-chain), and type 2 RIP consists of A-chain and B-chain (lectin-like domain). Multiple copies of RIP genes were observed in the Cucurbitaceae genome, but more paralogous genes, including both genes encoding A-chain (18 genes) and B-chain (8 genes), were present in the bitter gourd scaffolds than other cucurbit genomes (Supplementary Table S13). These bitter gourd RIP genes were clustered in six scaffolds, which were non-syntenic regions to other cucurbits genomes (Supplementary Table S15). Biological functions of RIPs in plants were not always well elucidated, but were possibly involved in the defense system against pathogenic fungi or bacteria via rRNA cleavage.^32^^,^^33^ On the other side, bitter gourd RIPs were well studied as a possible medically effective ingredient.^34^. Alpha-momorcharin and MAP30 were type-1 RIPs isolated from bitter gourd, and shown to have anti-viral and -tumor activity in mammalian cells.^35^^,^^36^ When these RIPs were used to treat HIV-infected cells, inhibition of viral replication was observed^36^ and additionally, MAP30 also inhibited integrase acitivty of HIV and topologically inactivated viral DNA^37^. Against tumor cells, it was demonstrated that both alpha-momorcharin and MAP30-induced apoptosis.^35^ Particularly, MAP30 caused little damage to normal cells,^38^ whereas cytotoxicity to normal liver cells was observed in alpha-momorcharin.^39^ Therefore, the clinical application of MAP30 was expected in cancers or protection for viral infection. Similarly, trichosanthin (TCS) from *Trichosanthes kirilowii*^40^ and balsamin from *Momordica balsamina*^41^ are known to have anti-viral activity. Alignment of amino acid sequences of balsamin showed that it was identical to MAP30 (data not shown). Phylogenetic analysis of TCS and the A-chain of RIPs in bitter gourd indicated TCS was closely related to alpha-momorcharin (Fig. 1). Although the biological functions of these trypsin inhibitors and RIPs are still unknown in bitter gourd plants, multiplication of genes for these proteins is unique to the bitter gourd genome, which might characterize bitter gourd as a medicinal plant.

### 3.5. Mapping of RAD-seq tags

We previously performed RAD-seq analysis to identify DNA polymorphisms between the two bitter gourd lines (OHB95-1A and OHB61-5).^12^ In this study, the RAD-seq tag sequences, represented by 80-base sequences from the *Ase*I-digested ends of genomic DNA, were mapped to the OHB3-1 scaffolds as reference sequences for finding polymorphic loci at unique positions in the genome. As shown in Supplementary Table S16, ∼90% of applied tags were mapped to unique positions of the OHB3-1 genome, whereas the other tags matched multiple loci on the genome. These uniquely mapped tags were distributed in 80% of the assembled scaffolds (Supplementary Table S16). Firstly, the RAD-tag mapping analysis determined the mismatches or indels between OHB61-5 or OHB95-1A and reference sequences (OHB3-1). As shown in Supplementary Table S16, ∼3% of the uniquely reference-mapped tags in each inbred line contained mismatches and/or indels, representing an average frequency of these polymorphic loci as once per 2.7 kb in the bitter gourd genome. In addition, based on the tag-mapping results, putative heterozygous loci in each parent line were identified as described in the method, and 1,279 and 884 loci were predicted to be heterozygous in OHB95-1A and OHB61-5, respectively (Supplementary Table S16). The frequency of these putative heterozygous loci was < 1% of the uniquely tag-mapped loci, implying low heterozygosity in these inbred lines.

### 3.6. Linkage map construction using RAD-seq markers

From the RAD-seq results of two inbred lines and tag mapping to scaffold sequences of OHB3-1 as a reference, bi-allelic tags at polymorphic loci between OHB61-5 and OHB95-1A could be identified as described in Supplementary method. Totally, 1,507 pairs (loci) of bi-allelic tags polymorphic between the two parental lines were identified and employed as co-dominant markers in linkage mapping analyses (Supplementary Table S17). An F_2_ population derived from OHB61-5 × OHB95-1A was previously developed^12^ and employed to generate a linkage map. Genomic DNA from 97 F_2_ individuals was used in RAD-seq analysis using *Ase*I, resulting in an average of 8,005,294 tags per an F_2_ plant. Genotypes of 1,507 bi-allelic tag markers in each F_2_ plant were determined based on the presence or absence of the allelic tag sequence. According to the genotype data of 1,507 markers in 97 plants, linkage positions of analyzed markers were determined using JoinMap4.1 software. At a cut-off LOD score = 7.0, 1,423 markers were separated into 11 linkage groups (Supplementary Table S18), which corresponded to the number of chromosomes in bitter gourd. The order of these markers in each linkage group was determined by calculating the genetic distances among markers, and a linkage map, encompassing 3,426 cM, was constructed (Supplementary Fig. S5). In accordance with the positions of the analyzed markers (bi-allelic tags) in the reference genome sequence, 255 scaffolds could be anchored to the constructed linkage map (Supplementary Table S18). Although analyzed RAD-seq tags were located in 80% of the assembled scaffolds, only 255 scaffolds (25%) were assigned on the linkage map, indicating that the frequency of bi-allelic tags were limited. Positions of RAD tag markers in each scaffold almost corresponded to their order in the linkage map, although inconsistency was occasionally observed in 473 loci. Nevertheless, the present result was far from developing pseudomolecules in the bitter gourd genome, since many scaffolds remained to be assigned on this linkage map. Considering the limited genetic diversity in bitter gourd lines, it might be difficult to increase marker density; thus, improving scaffold length by using a different assembly procedure and/or long read sequencing method such as PacBio, is necessary for completing its genome sequence analysis.

### 3.7. Orthologous genes for sex determination

Cucurbit crops are suitable models for elucidating sex determination in monoecious plant species.^42^ Since genes for sex determination have been identified in melon and cucumber, their orthologous genes were further explored in other Cucurbitaceae crops by BLAST searching, including bitter gourd. *CmAcs11*, encoding ACC synthase, responsible for female flower determination in melon.^43^ An additional ACC synthase gene, *CmAcs-*7,^44^ was also shown to regulate unisexual flower development in melon and putative orthologous genes were identified in related species. Amino acid sequences of these ACC synthases in cucurbits (Supplementary Table S12) were aligned and a phylogenetic tree was created including genes in *Arabidopsis thaliana* as an outgroup (Supplementary Fig. S6A). CmAcs-7 and its homologous proteins in cucurbits were phylogenetically distant from CmAcs11 and its homologs, suggesting the differentiation of these two ACC synthases. According to this analysis, the MOMC3_649 in bitter gourd (Supplementary Fig. S6A) was presumed to be an ortholog of CmAcs11. Two proteins similar to CmAcs-7 (MOMC46_189, MOMC518_1) were found in bitter gourd (Supplementary Fig. S6A), and grouped in the same clade in the phylogenetic tree.

As the gene for male determination, *CmWip1*, encoding the zinc-finger domain protein, was identified in melon.^45^ Its putative orthologous genes were also searched in the genome of other Cucurbitaceae species and their phylogenetic relationship was also analyzed based on amino acid sequences of their encoded proteins as described above (Supplementary Fig. S6B). In this analysis, MOMC52-27 in bitter gourd (Supplementary Fig. S6B) was assumed to be an ortholog of CmWip1. These results showed that orthologous genes for known sex determination genes in melon or cucumber were also present in bitter gourd.

RT-PCR analysis (Supplementary Fig. S7) demonstrated that *MOMC518_1g*(encoding CmAcs-7-like protein) and *MOMC3_649g* (encoding CmAcs11-like protein) were expressed in female flowers of bitter gourd plants more preferentially than male flower buds. This was in agreement with previous observations in melon or cucumbers, where both *ACS11* and *ACS-7* or *ACS2* were highly expressed in female flower buds.^43^ Another gene encoding CmAcs-7-like protein in bitter gourd, *MOMC46_89g*, did not show specific expression in flower buds. Expression of the genes for *MOMC52_27g* (encoding CmWip1-like protein) was confirmed in flower buds, but significant differential expression among analyzed tissues was not observed, which was inconsistent with its male flower-specific expression in melon or cucumber.^45^ As shown by Switzenberg *et al.*^46^ in melon, specific expression of *ACS* gene in petal and stamen induced alternation of sex phenotype by ethylene production. Therefore, to elucidate functions in the sex determination of candidate genes in bitter gourd, their spatiotemporal expression patterns should be analyzed in flower buds.

In *Momordica*, dioecious species, such as *M. dioica* or *M. cochinchinensis*, are also known. According to molecular phylogenetics, an African ancestor of *Momordica* species was predicted to be dioecious, and several conversions between dioecy and monoecy have occurred during its speciation and distribution from Africa to Asia.^47^ In *M. dioica*, it was suggested that ethylene was responsible for sex determination,^48^ although sex determination genes were undetermined. Therefore, molecular genetic studies of sex determination in *Momordica* species, including bitter gourd, will reveal the evolution of dioecy and monoecy. Boualem *et al.*, succeeded in establishing dioecious mating system in melon by combining alleles of *CmAcs11* and *CmWip1*.^43^^,^^47^ However, it was difficult to consider that these two independent genes had concerned to frequent conversion between monoecy and dioecy in *Momordica* species.

## 5. Data availability

The DRA accession number for the reads generated through Illumina genome sequencing is DRA004548. The scaffold sequences are available under the accession numbers BDCS01000001–BDCS01001052 (1052 entries).

## Supplementary Material

Supplementary Data
