# Supplementary material for: Draft genome sequence of bitter gourd (Momordica charantia), a vegetable and medicinal plant in tropical and subtropical regions
Source: DNA Res. 2016 Dec 17;24(1):51–8. doi: 10.1093/dnares/dsw047 (PMC5381343; doi:10.1093/dnares/dsw047)
Supplement: Supplementary Data [file dsw047_Supp.zip › Suppl Fig S1.pdf]

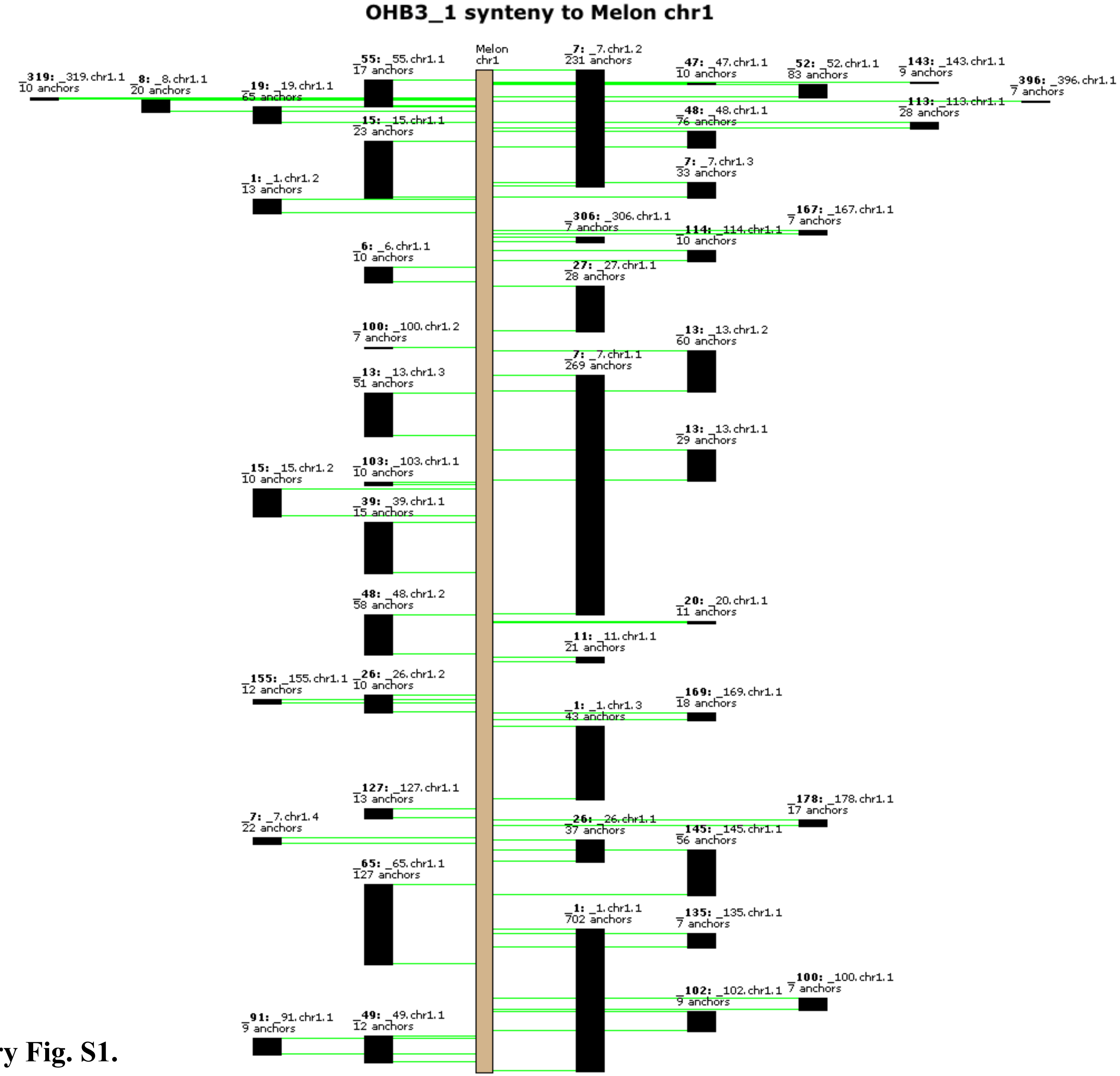

Supplementary Fig. S1.

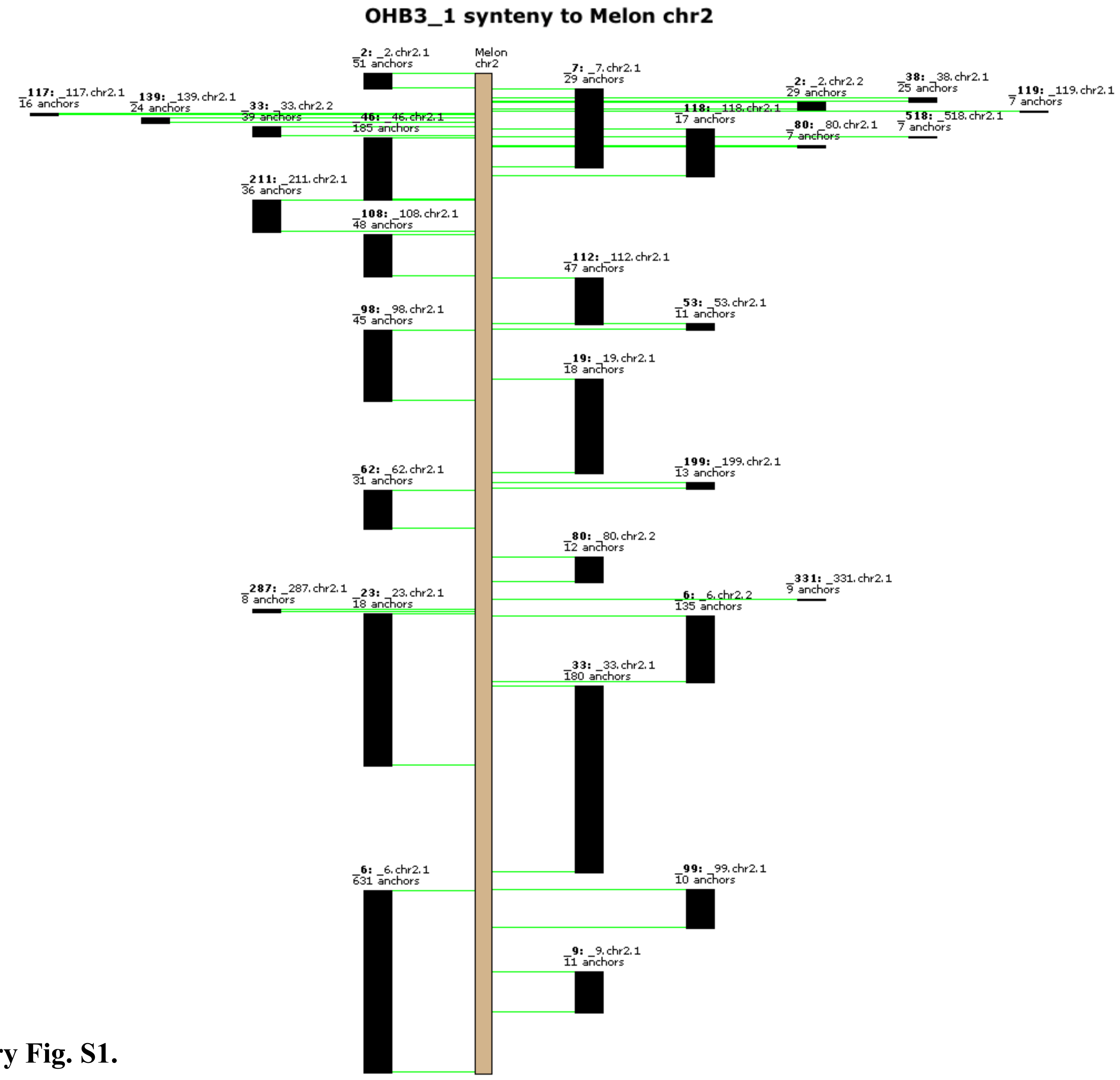

Supplementary Fig. S1.

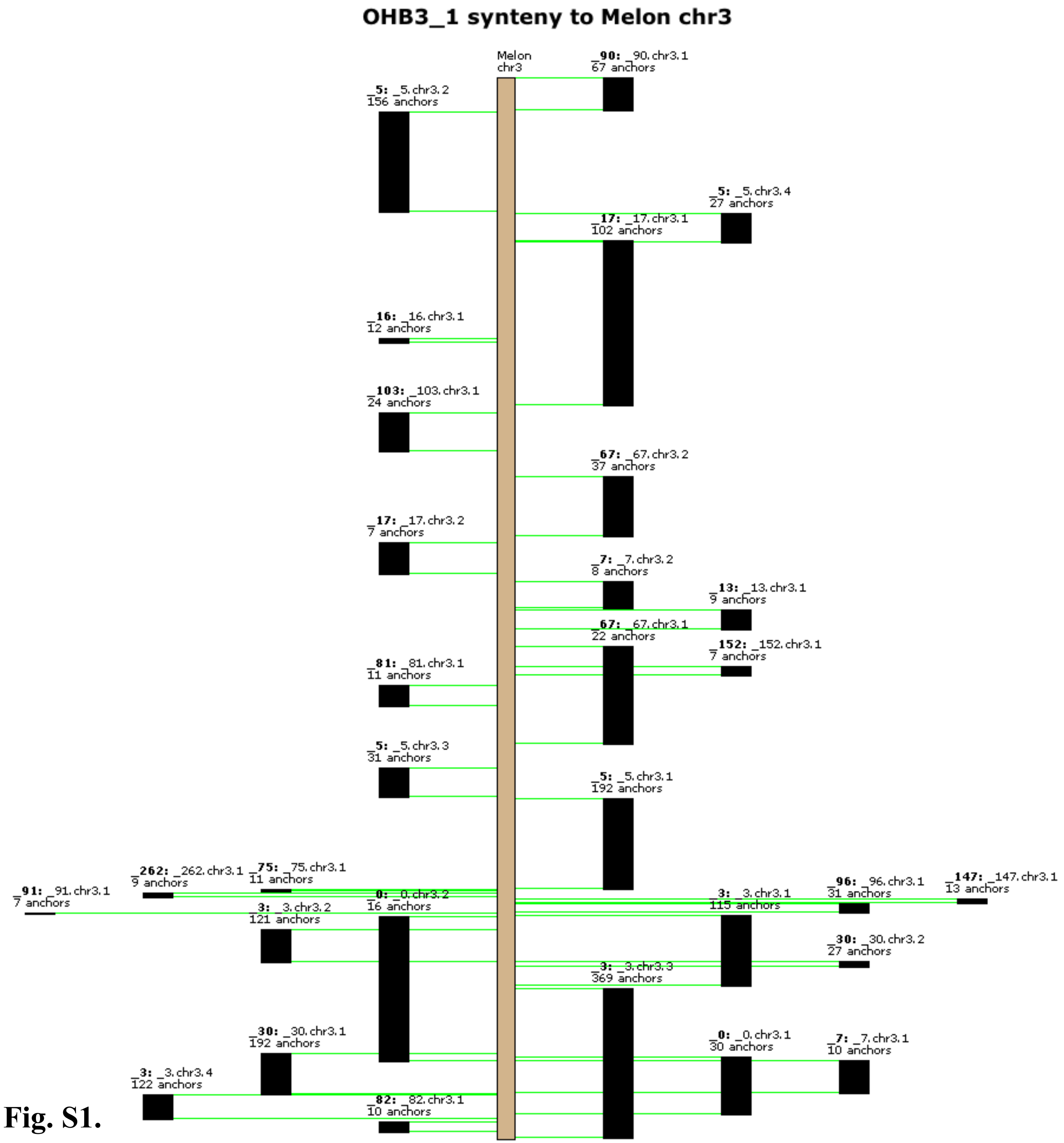

Supplementary Fig. S1.

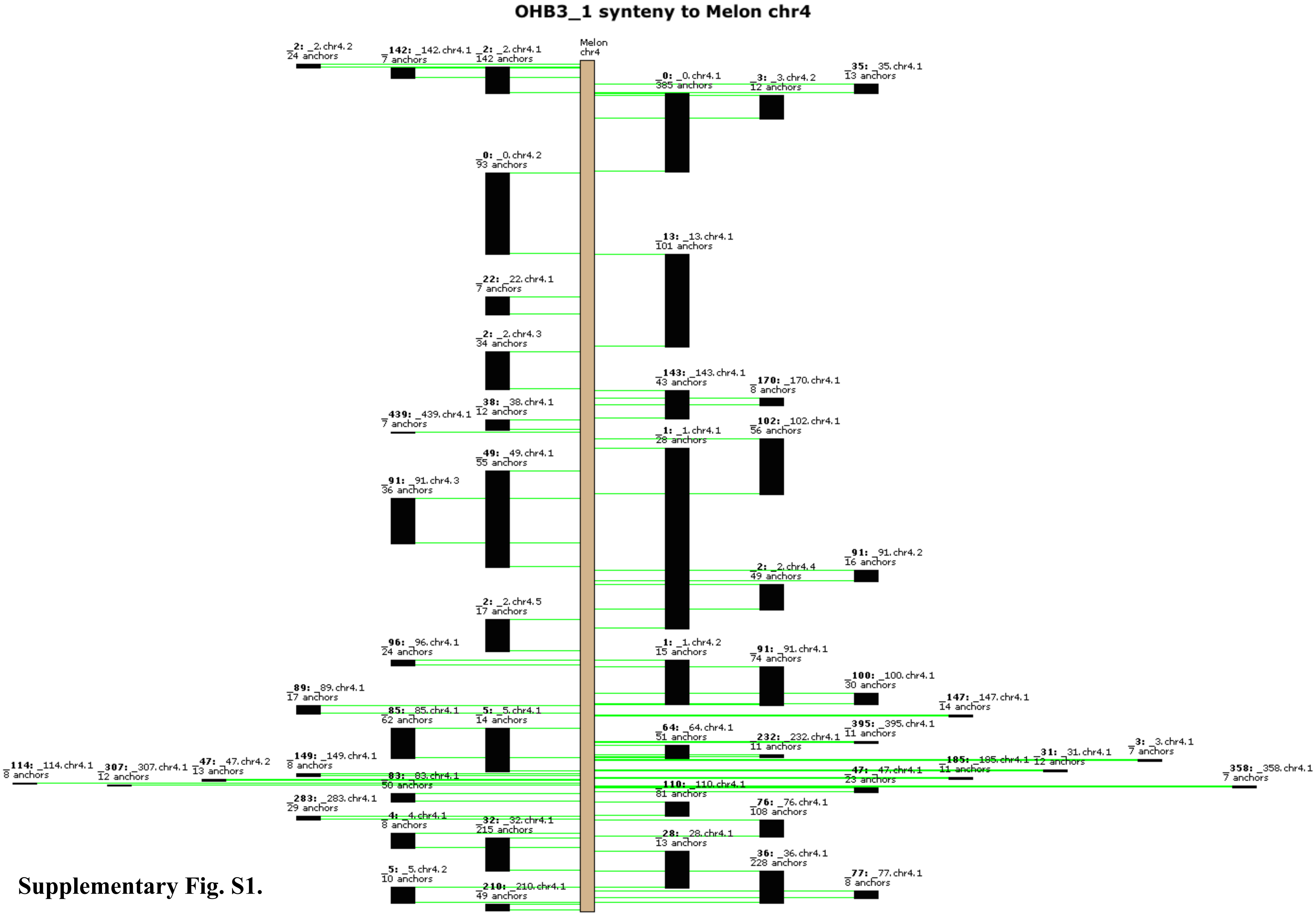

Supplementary Fig. S1.

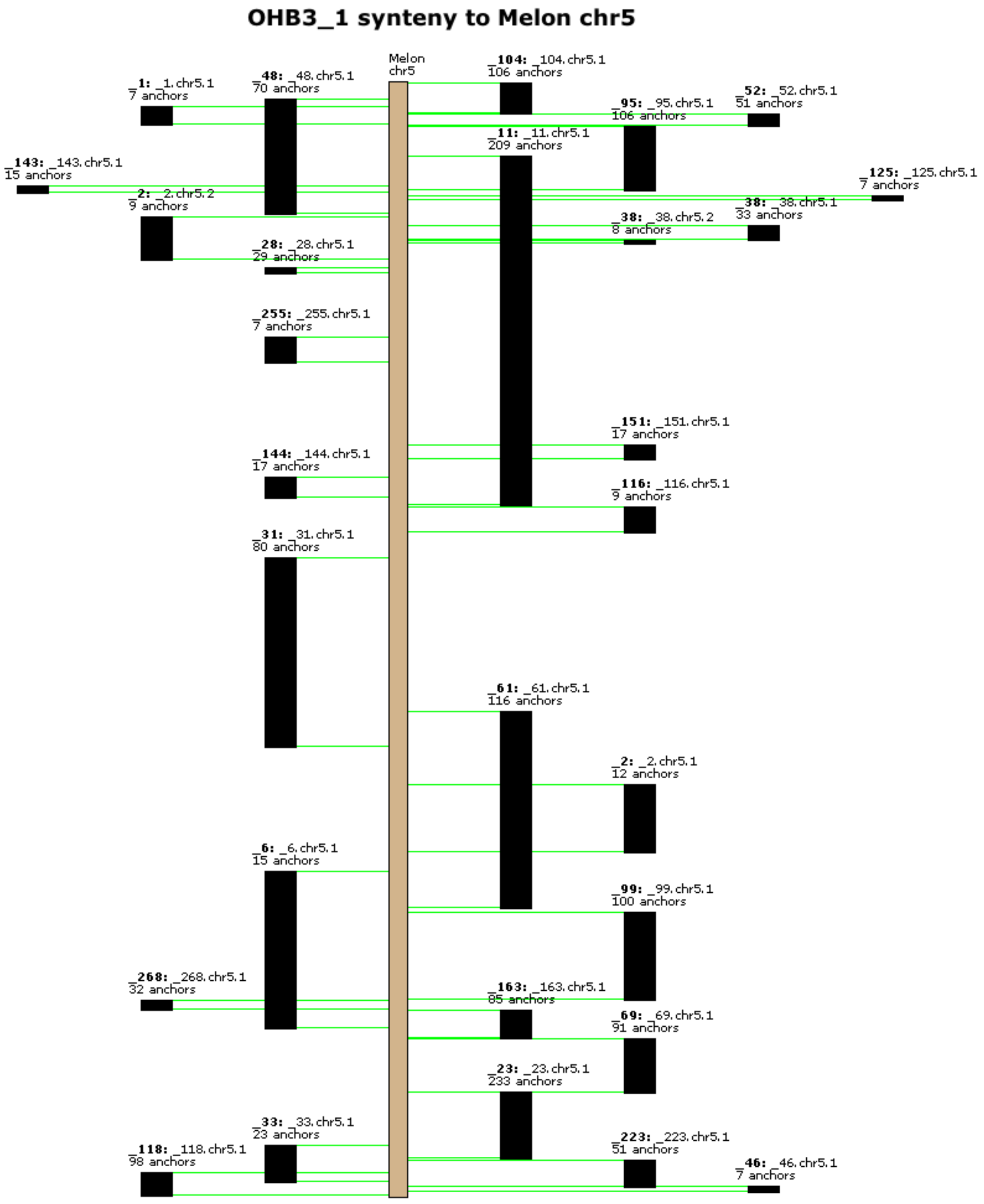

Supplementary Fig. S1.

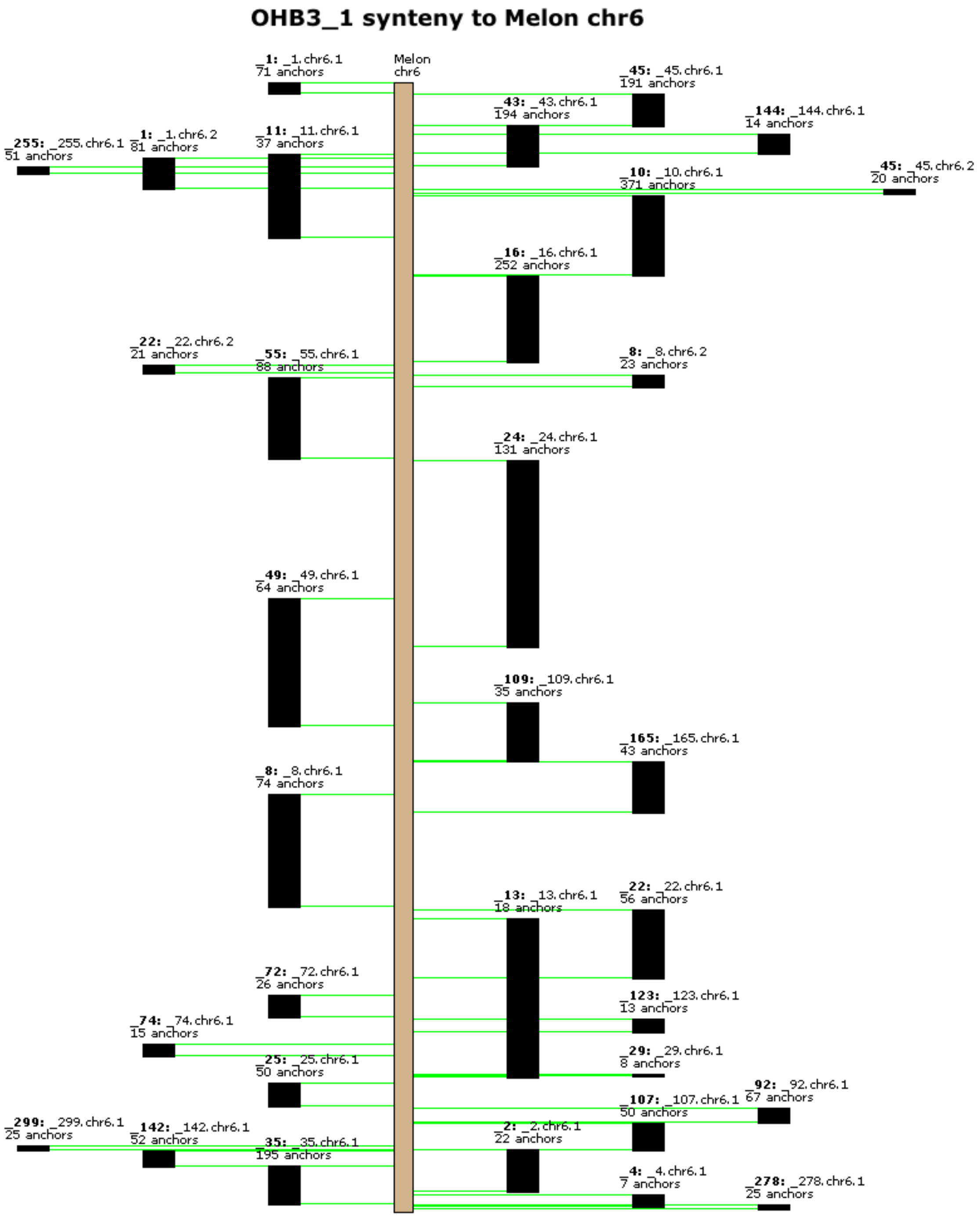

Supplementary Fig. S1.

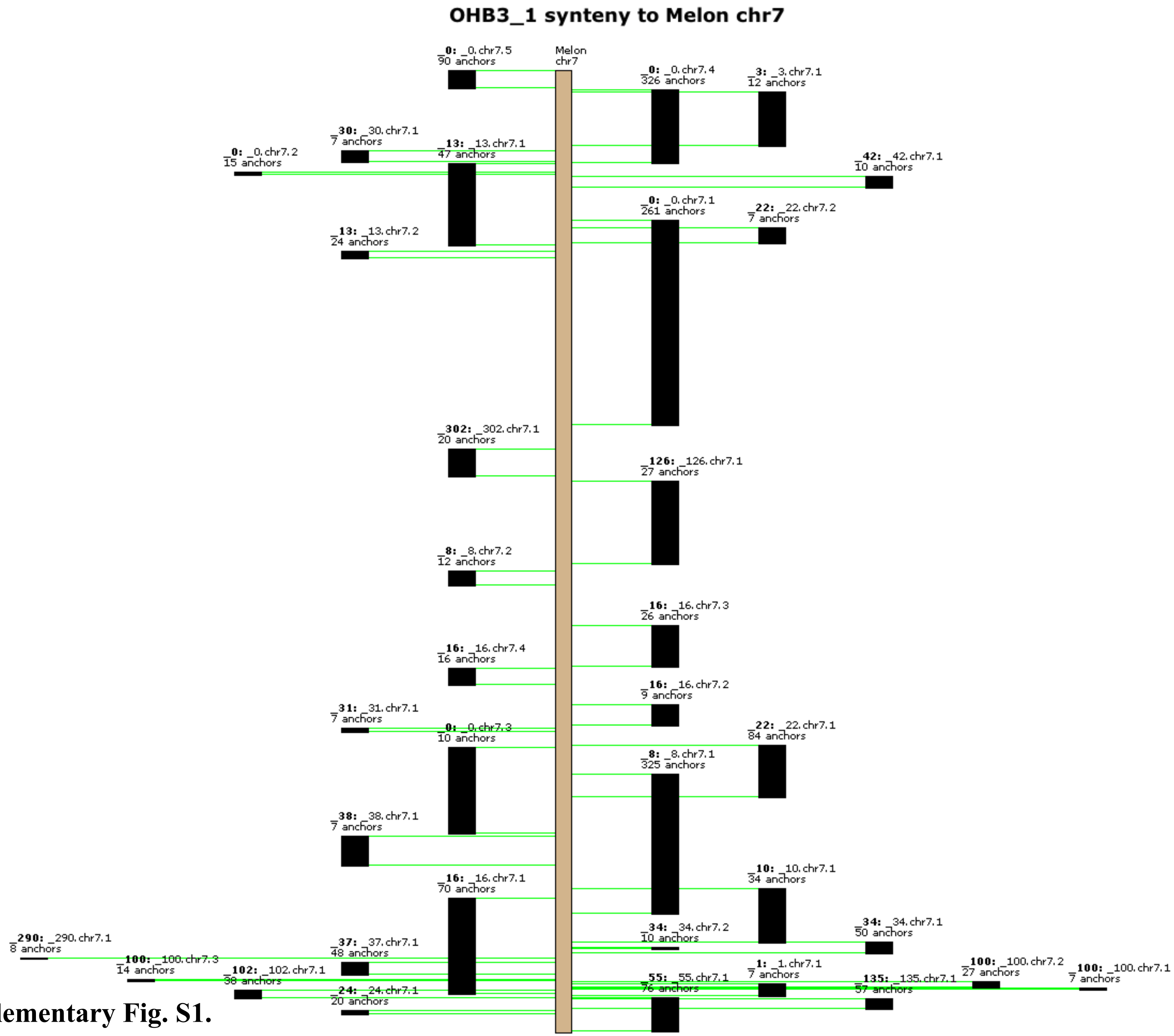

Supplementary Fig. S1.

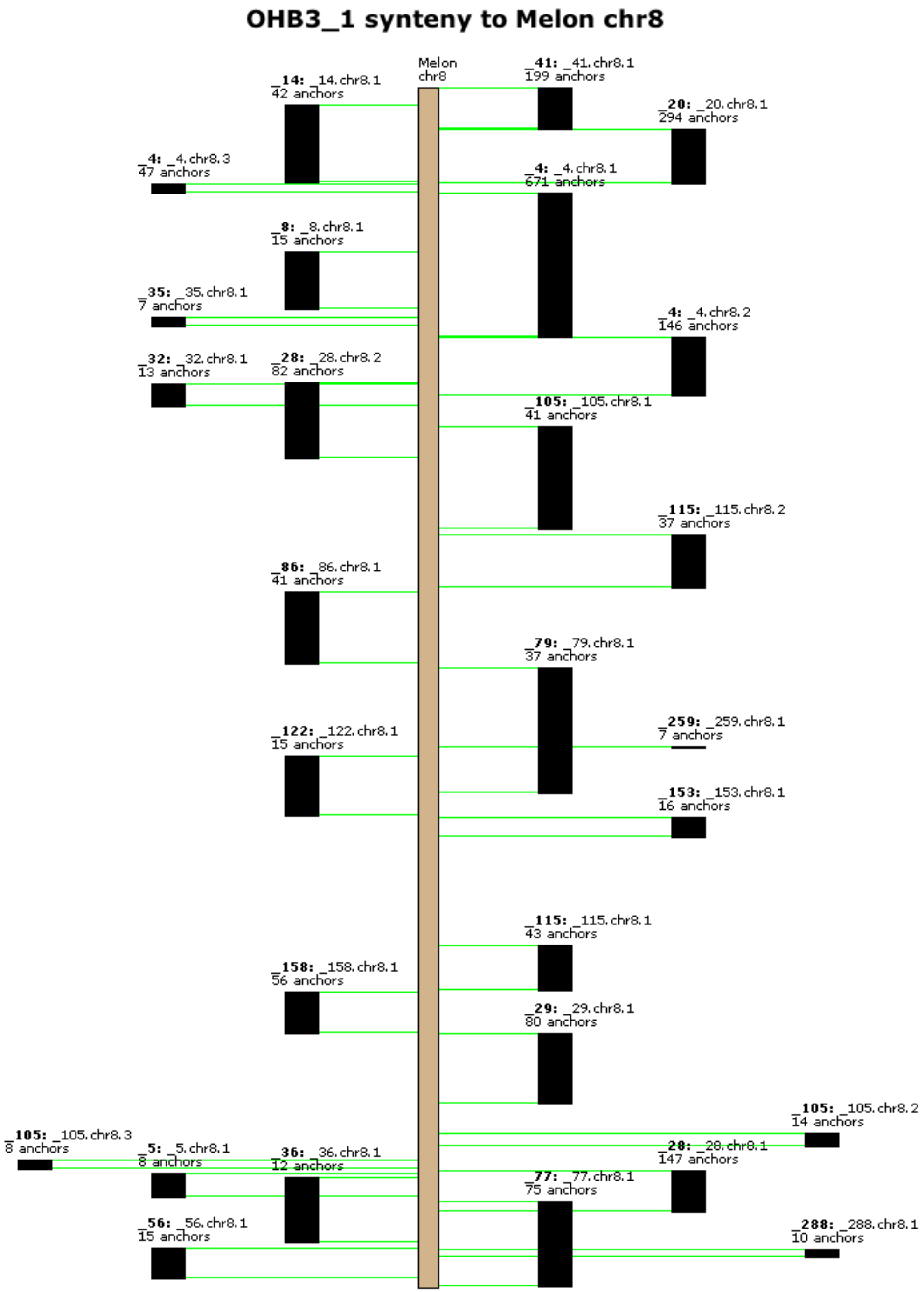

Supplementary Fig. S1.

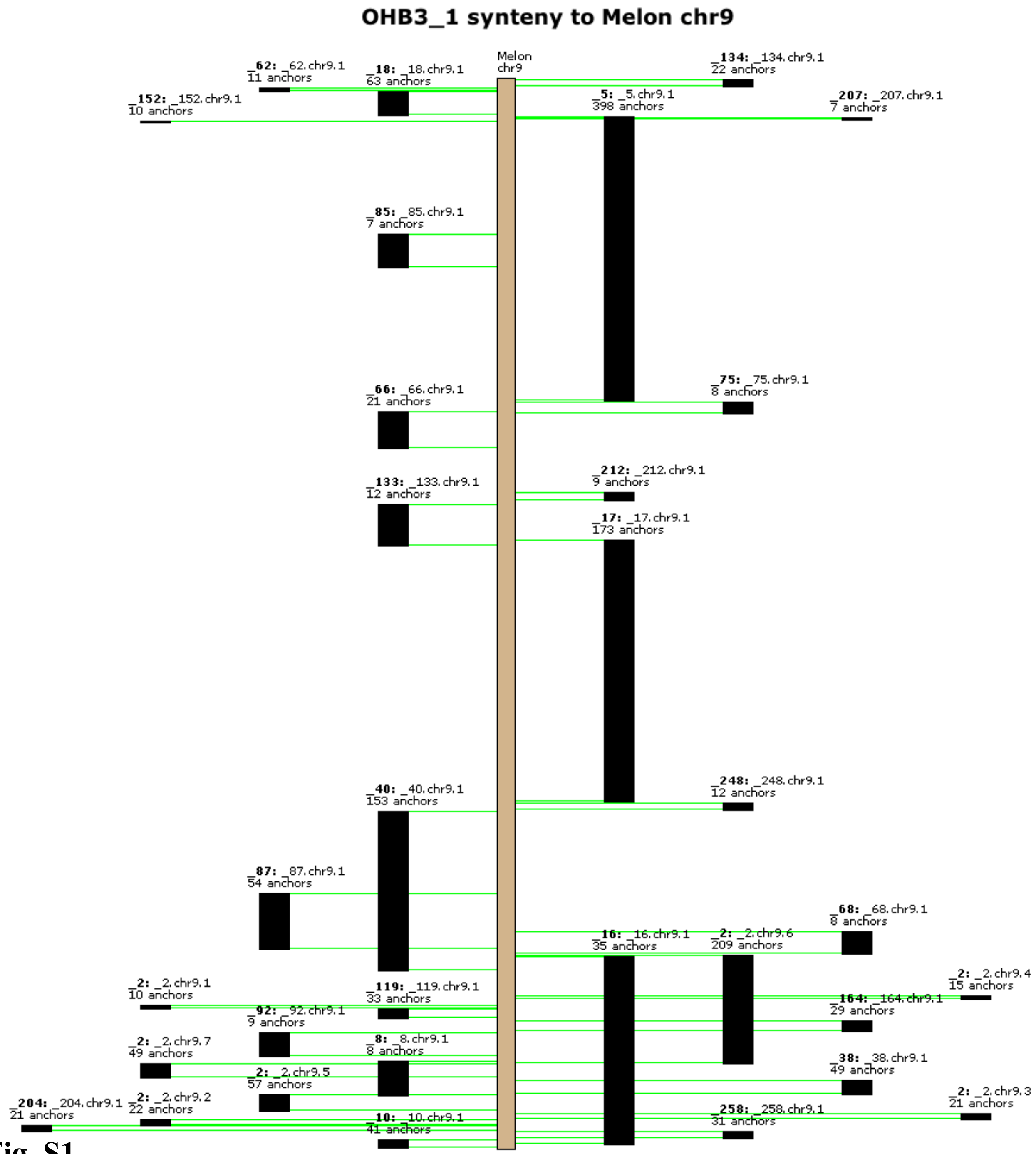

Supplementary Fig. S1.

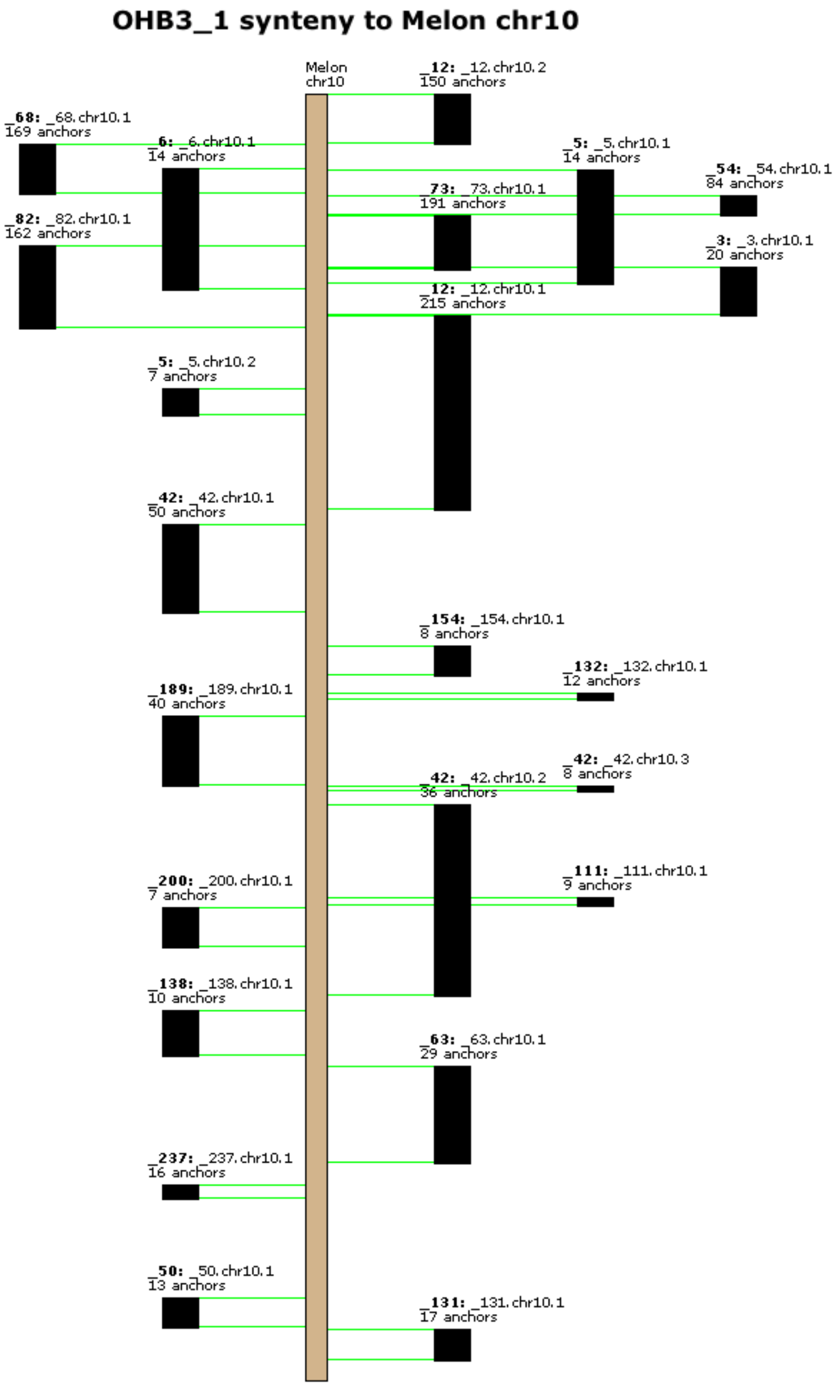

Supplementary Fig. S1.

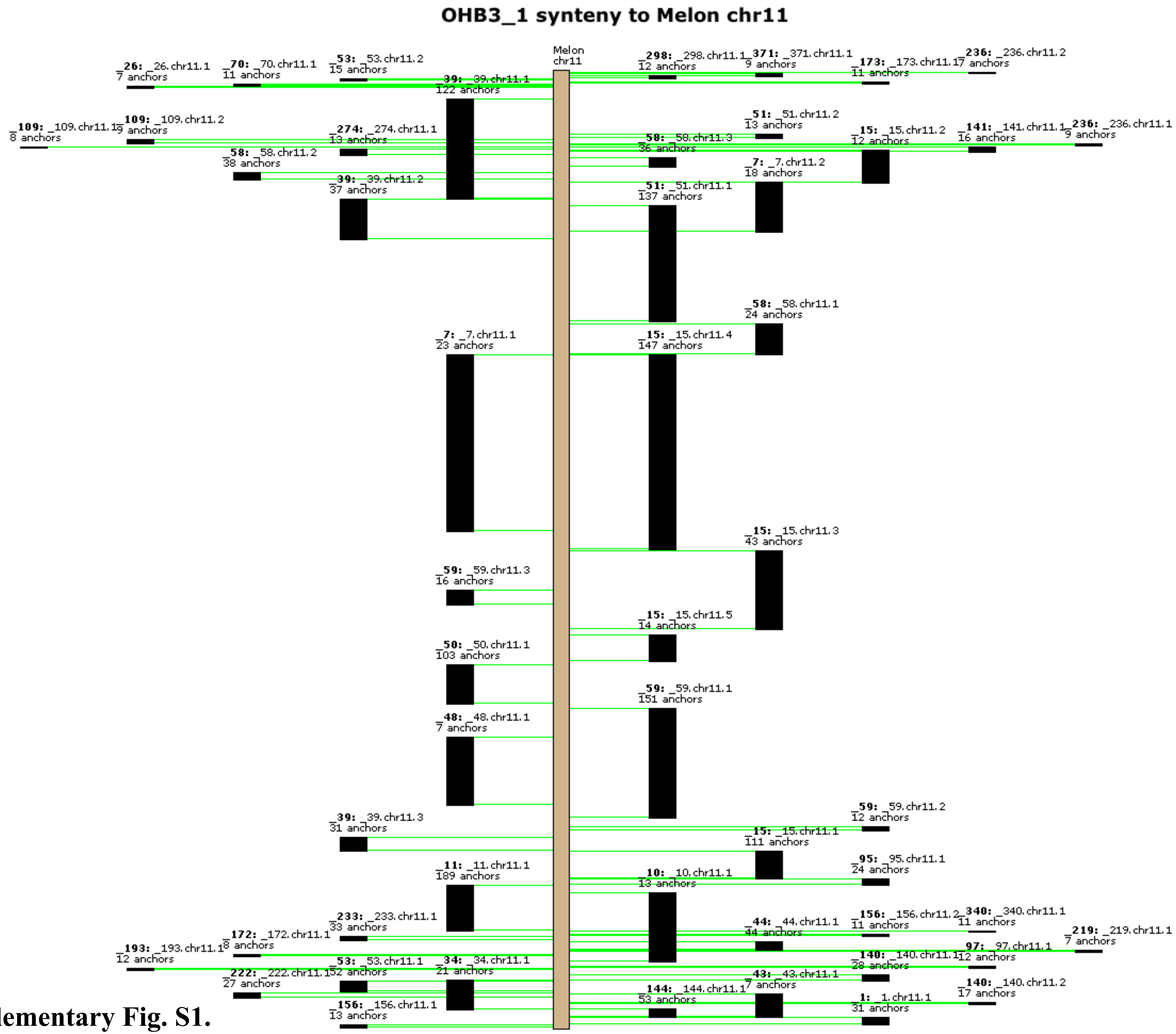

Supplementary Fig. S1.

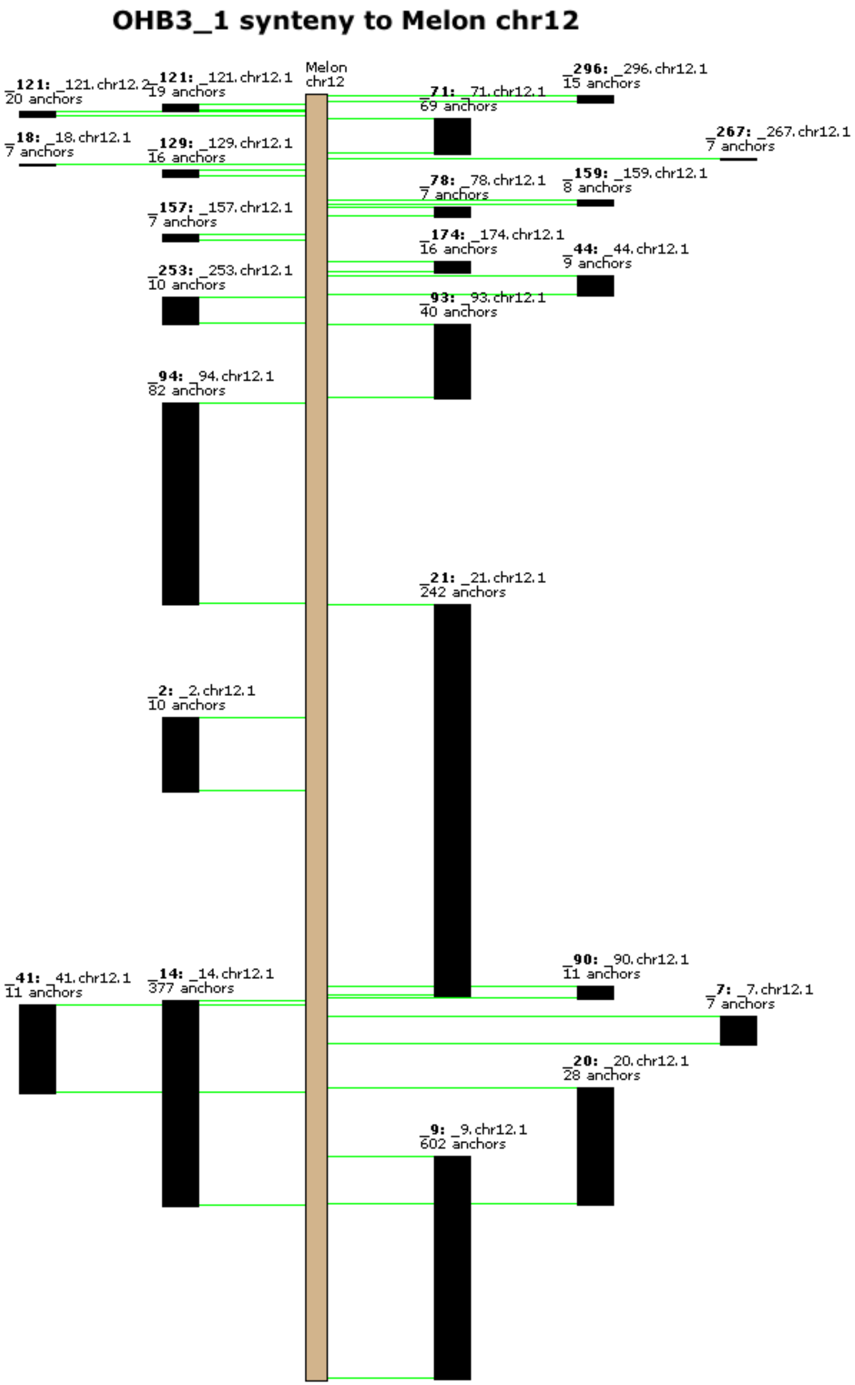

Supplementary Fig. S1.

Supplementary Fig. S1. Synteny block view of bitter gourd scaffolds to the melon genome sequence.

Synteny blocks between bitter gourd (OHB3-1) scaffolds to pseudomolecule sequences of melon were identified and visualized using SyMap4.2. Each chromosome (pseudomolecule) was indicated as a vertical beige-colored bar. Black bars around each chromosome were mapped synteny blocks of bitter gourd scaffolds. Above each synteny block bar, the scaffold ID, synteny block ID, and number of constituted anchors were indicated.
