# Supplementary material for: Draft genome sequence of bitter gourd (Momordica charantia), a vegetable and medicinal plant in tropical and subtropical regions
Source: DNA Res. 2016 Dec 17;24(1):51–8. doi: 10.1093/dnares/dsw047 (PMC5381343; doi:10.1093/dnares/dsw047)
Supplement: Supplementary Data [file dsw047_Supp.zip › Suppl Fig S4.pdf]

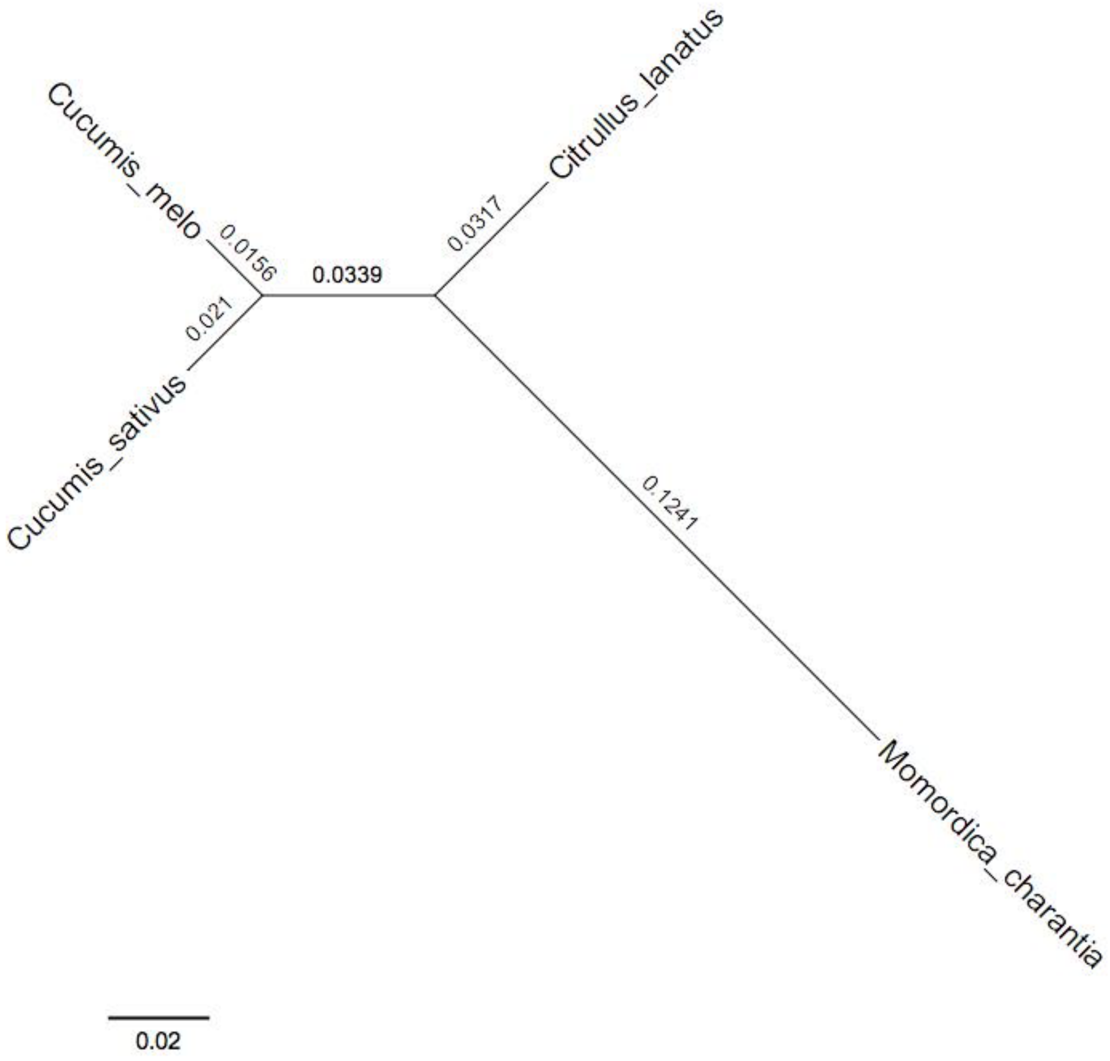

Supplementary Fig. S4. Phylogenetic relationship among Cucurbitaceae species based on deduced amino acid sequences of 69 selected loci of orthologous genes.

Amino acid sequences of encoded proteins for selected genes (Supplementary Table S11, S12) were concatenated and the best substitution model was selected by Aminosan program(Supplementary Table S11). Then, based on the calculated model, a maximum likelihood phylogenetic tree was generated by RAxML and FigTree as described in Supplementary method. Evolutionary distance (substitution per a site) was indicated on each branch and under the bar.
