# Supplementary material for: Draft genome sequence of bitter gourd (Momordica charantia), a vegetable and medicinal plant in tropical and subtropical regions
Source: DNA Res. 2016 Dec 17;24(1):51–8. doi: 10.1093/dnares/dsw047 (PMC5381343; doi:10.1093/dnares/dsw047)
Supplement: Supplementary Data [file dsw047_Supp.zip › Suppl Fig S6.pdf]

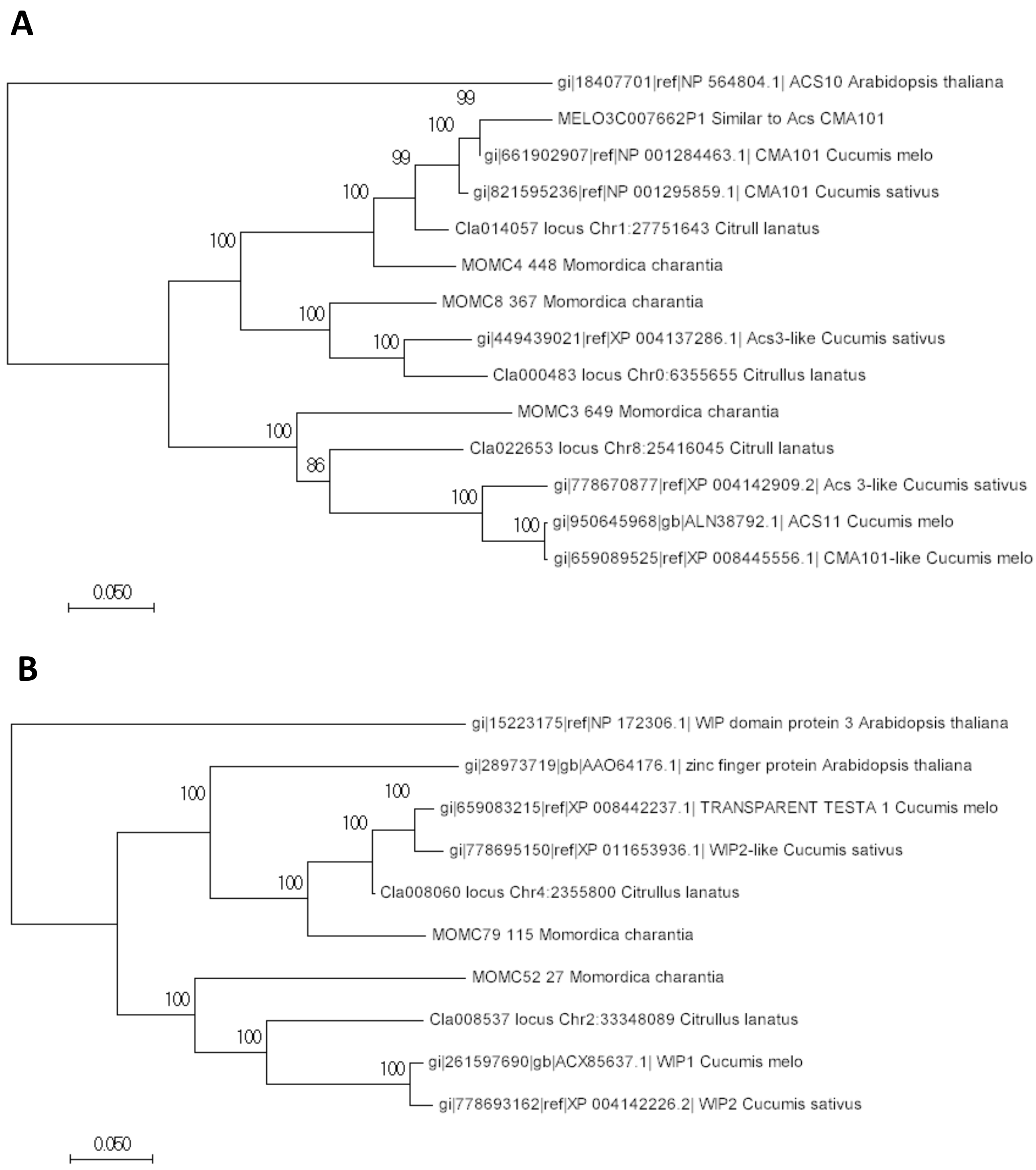

Supplementary Fig. S6. Phylogenic analysis of sex determination-related genes in Cucurbitaceae

Neighbor-joining tree based on amino acid sequences of ACC synthases (A) and Wip1(B) from Cucurbitaceae plants and *Arabidopsis thaliana* as an outgroup. The bootstrap values (500 replicates) not less than 50 are shown next to the branches. The scale bar corresponds to 0.1 substitutions per site. Phylogenetic tree was developed using MEGA7.0.18.
