# Supplementary material for: Draft genome sequence of bitter gourd (Momordica charantia), a vegetable and medicinal plant in tropical and subtropical regions
Source: DNA Res. 2016 Dec 17;24(1):51–8. doi: 10.1093/dnares/dsw047 (PMC5381343; doi:10.1093/dnares/dsw047)
Supplement: Supplementary Data [file dsw047_Supp.zip › Suppl Fig S7.pdf]

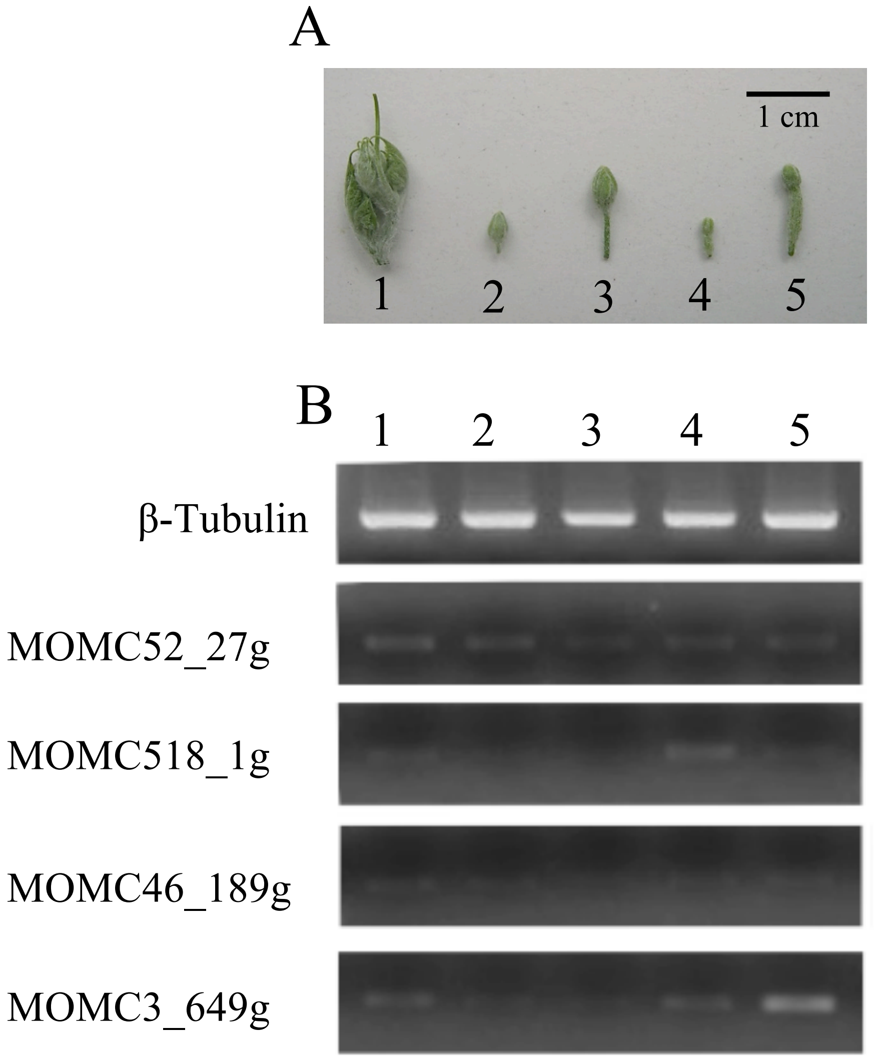

Supplementary Fig. S7. Expression analysis of orthologous genes for sex determination in bitter gourd.

A. Representatives of the apical meristem and flower buds used in this analysis. 1, apical meristem; 2 and 3, male flower buds; and 4 and 5, female flower buds. B. RT-PCR. As a control, ß-tubulin was amplified. Numbers in B correspond to those in A.
