# Supplementary material for: Draft genome sequence of bitter gourd (Momordica charantia), a vegetable and medicinal plant in tropical and subtropical regions
Source: DNA Res. 2016 Dec 17;24(1):51–8. doi: 10.1093/dnares/dsw047 (PMC5381343; doi:10.1093/dnares/dsw047)
Supplement: Supplementary Data [file dsw047_Supp.zip › Suppl method_Oct21.pdf]

Supplementary method

Gene prediction and annotation

Gene prediction analysis in the bitter gourd scaffold sequences was carried out using *ab initio* prediction by FGENESH software ver 3.1.1 (Softberry)

Using specific gene finding parameters for *Arabidopsis thaliana* and a standard translation codon table, positions of coding sequences (CDSs) in the scaffold sequences were predicted and a region including a set of CDSs initiated from a start codon and ended at a stop codon, was defined as a “predicted gene” in this analysis. In addition to CDS, the transcription start or polyadenylation site of each predicted gene was predicted. Simultaneously, the amino acid sequence of encoded proteins was also translated from the CDS of each predicted gene based on the standard translation codon table by FGENESH.

For annotating predicted genes, encoded protein sequences were applied to the BLASTP search against the non-redundant (NR) protein database in NCBI (ftp://ftp.ncbi.nlm.nih.gov/blast/db/) and UniProtKB/Swiss-Prot database (http://www.uniprot.org), respectively. The threshold for BLAST searching was determined as an e-value < 0.001 and only the top hit protein in each search was listed. In the analysis, all the applications were implemented, but the results of Pfam, SMART, ProDom, and PRINTS, which generally represented the functional annotation of analyzed proteins, were summarized.

Comparative analysis of genomes among Cucurbitaceae species

Scaffold or pseudomolecule sequence files (fasta format) and predicted gene annotation file (gff3

format) for each species were uploaded in the SyMap4.2. In the SyMap, anchors, which connect two genomes (or scaffold) via annotated (predicted) genes, were identified using sequence alignment based on the MUMmer program (http://mummer.sourceforge.net/) at PROmer mode and filtered using a reciprocal-top-two filter. As a synteny block, a region containing at least seven anchors was defined.

Conserved gene determination and phylogenetic analysis

By comparing a list of anchors analyzed by the SyMap program, genes showing conserved synteny among all four Cucurbitaceae species were identified. Amino acid sequences of their encoded proteins were applied to a BLASTP search against encoded proteins of all the predicted genes in each genome. Genes showing similarity to multiple genes in the genome at the amino acid level were excluded, and only the non-redundant (unique) genes were selected. Genes, unique in the genome of each species and conserved among the four species, were defined as orthologous genes. For 69 selected loci of orthologous genes, encoded protein sequences were individually aligned among four species by clustalW (gap opening penalty 10, gap extension penalty 0.2, Gonnet as protein weight matrix, delay divergent cutoff 30%) using MEGA7.0.18. For concatenation of amino acid sequences of 69 genes in each species and substitution model selection, alignment files were applied to the Aminosan program (https://www.fifthdimension.jp/products/aminosan/)[20]. For the analysis, a partitioned model of concatenated sequences was employed and the output option for RAxML was specified. Following the script in the output file (whole_AIC_partitionedequalmeanrate_codonpartitionedequalmeanrate_shotgunresearch.bat and whole_AIC_partitionedequalmeanrate_codonpartitionedequalmeanrate_bootstrap.bat) from the

Aminosan program, the RAxML (http://sco.h-its.org/exelixis/web/software/raxml/index.html) was run to calculate the maximum likelihood trees. The calculated trees and bootstrap files were analyzed using pgsumtree command in Phylogears2 (https://www.fifthdimension.jp/products/phylogears/), which produced a newick format file and its phylogenetic tree was drawn using FigTree version 1.4.2 (http://tree.bio.ed.ac.uk/software/figtree/).

Reference mapping of RAD-seq tags

Tag sequences showing more than 20 counts in either parent line (OHB61-5 or OHB95-1A) were employed in further analysis. These tag sequences (80 bp) were mapped to the scaffold sequences of OHB3-1 as "reference sequences" using BWA version 0.6.1. After reference sequence data were converted by an index command with the a-option, read mapping was conducted using the aln and samse command with default settings (allowed < 3% mismatches and indels) for the output SAM format file. Using the uniq option, multiple reference-mapped tags were eliminated. It was determined that a tag was mapped at a unique position in the reference sequences when its mapping quality score in the SAM formatted data was 37. This tag was defined as a "uniquely mapped tag" in this study, and employed for identifying polymorphic or heterozygous loci. Any sequence differences between reference sequences and uniquely mapped tag sequences were considered to be polymorphisms between OHB3-1 (reference sequences) and OHB95-1A or OHB61-5. Heterozygous loci in individual parent line were defined when two independent tags with different sequences from the same parent line (OHB61-5 or OHB91-5A) were mapped at the identical position of reference sequences. For identifying polymorphic tags (loci) between OHB61-5 and OHB95-1A, specifically observed tags in either inbred line were found by comparing uniquely reference-mapped tags

between two inbred lines. When OHB61-5- and OHB95-1A-specific tags were uniquely mapped in the same position of reference sequences, these tags represented alleles (bi-allelic tag) at a polymorphic locus.

Comparative analysis of orthologous and paralogous genes

In unique genes found in the bitter gourd scaffolds, genes encoding proteins with the Proteinase inhibitor I7 or Proteinase inhibitor I13 domain, or similar to known trypsin-inhibitor protein sequences were defined as putative trypsin inhibitor genes. Similarly, genes encoding proteins with the Ribosome-inactivating protein or Ricin B lectin domain were classified as chain-A or chain-B subunit genes for ribosome inactivating proteins. Their homologous genes in melon, cucumber, or watermelon genomes were identified by BLAST searches against encoded amino acid sequences of predicted genes in each genome.

For exploring homologues of sex determination genes, amino acid sequences of CmWip1 (ACX85637.1), CmAcs11 (ALN38792.1), and CmAcs-7 (ACG70850.1) were queried through a BLAST search against predicted gene sequences in cucumber, melon, watermelon, and bitter gourd.

Amino acid sequences of selected genes were aligned by clustalW (gap opening penalty 10, gap extension penalty 0.2, Gonnet as protein weight matrix, delay divergent cutoff 30%) using MEGA7.0.18. Using the same software, phylogenetic trees were constructed using the Neighbor-Joining (NJ) method and inferred from 500 replicates of bootstrap tests, and the evolutionary distances were computed using the p-distance method and gaps or mismatches were treated by pairwise deletion.

RT-PCR analysis

The total RNA was extracted from the apical meristem, 3–4 mm of the male and female flower buds, and 10–15 mm of the male and female flower buds of the bitter gourd F_1 cultivar ‘Shiokaze’ using the RNeasy Plant Mini Kit (Qiagen, Valencia, CA, USA). Three micrograms of total RNA were applied to cDNA synthesis using a PrimeScript™ II 1st strand cDNA Synthesis Kit (Takara Bio, Shiga, Japan). The oligo dT- adaptor primer was used for reverse transcription. Total RNA extraction and cDNA synthesis was carried out according to the manufacturer’s instructions. For RT-PCR, these cDNA samples were used as templates. As a control of gene expression, the ß-tubulin gene (Yang, et al., 2010*) was employed. As the primer set MOMC52_27_wip1_F (5′-GGACTTCAGAACCCTTCAGACA-3′), MOMC52_27_wip1_R (5′-GGAGTGAGTTGCATTGGCAT-3′), MOMC518_1_acs7_F (5′-GCATACTGAAGGAAGTGAAATTGA-3′), MOMC518_1_acs7_R (5′-TTAGATATTTGCTTCTTCCTTCTTC-3′), MOMC46_189_acs7_F (5′-ACTGCATTTTGAAGGAAGCCA-3′), MOMC46_189_acs7_R (5′-TCATCCTTTGAAGTCATCTCCT-3′), MOMC3_649_acs11_3_F (5′-GAACGCCGGAATCGGGTGCTTG-3′), MOMC3_649_acs11_2_R (5′-CGACGAATGTCTTGAGACGACTT-3′) were designed for amplification, respectively. PCR was performed using a 25-μL reaction volume with the DNA polymerase KOD FX (TOYOBO LIFE SCIENCE, Osaka, Japan). Amplifications were performed under the following conditions: preheating at 94°C for 2 min, followed by 32 cycles of amplification at 94°C for 10 sec, annealing at 55°C for 10 sec, and extension at 68°C for 1 min, and a final extension at 68°C for 5 min. PCR products were separated using 1.5% agarose gel electrophoresis.

* Yang, P., Li, X., Shipp, M.J., et al., E.B. 2010, Mining the bitter melon (*Momordica charantia* L.) seed transcriptome by 454 analysis of non-normalized and normalized cDNA populations for conjugated fatty acid metabolism related genes. *BMC Plant Biol.* **10**, 250.

Linkage map development

An RAD-seq analysis of 97 F_2 plants derived from OHB61-5 and OHB95-1A was carried out as described above. Based on analyzed RAD-seq data in individual F_2 plants, genotypes of bi-allelic tags as co-dominant markers were determined following a previously described method

Heterozygote genotypes were represented by the presence of both parental allele tags at a locus, whereas homozygote genotypes were represented by the presence of either the maternal or the paternal allelic tag at a locus. A linkage map was developed for the F_2 population in JoinMap4.1 (Kyazma)[22] using specific grouping and marker ordering with a maximum likelihood (ML) mapping algorithm with preset default parameter settings. A linkage group was defined as a group that consisted of more than 10 markers, and an appropriate logarithm of odds (LOD) score for linkage grouping was determined by evaluating the number of linkage groups in accordance with the increasing LOD score.
