# Supplementary material for: Draft genome sequence of bitter gourd (Momordica charantia), a vegetable and medicinal plant in tropical and subtropical regions
Source: DNA Res. 2016 Dec 17;24(1):51–8. doi: 10.1093/dnares/dsw047 (PMC5381343; doi:10.1093/dnares/dsw047)
Supplement: Supplementary Data [file dsw047_Supp.zip › Suppl Tab S3.pdf]

Supplementary Table S3. Sequence reads of bitter gourd (OHB3-1) genome for *de novo* assembly

Platform	Type of library	Insert size	Read information[c]	
			Read count	Total (bp)
MiSeq[a]	Paired-end	330 (bp)	28,471,244	6,554,590,527
HiSeq2500[b]	Mate-paired	2 (kbp)	91,333,824	7,772,739,816
		4 (kbp)	90,767,040	7,748,969,573
		6 (kbp)	87,007,574	7,486,535,396
		8 (kbp)	93,330,418	7,929,624,856

[a]Applying to 250 bp paired-end sequencing.

[b]Applying to 100 bp paired-end sequencing.

[c]FASTQ reads from the NGS platforms were filtered with the FASTX-Toolkit 0.0.13. (http://hannonlab.cshl.edu/fastx_toolkit/) using the following criteria: short reads were used only when nucleotides with a PHRED quality score 30 comprised 90% of the read.
