# Supplementary material for: Draft genome sequence of bitter gourd (Momordica charantia), a vegetable and medicinal plant in tropical and subtropical regions
Source: DNA Res. 2016 Dec 17;24(1):51–8. doi: 10.1093/dnares/dsw047 (PMC5381343; doi:10.1093/dnares/dsw047)
Supplement: Supplementary Data [file dsw047_Supp.zip › Suppl Tab S9.pdf]

Supplementary Table S9. Summary of synteny mapping of bitter gourd scaffolds to the genome sequence of cucumber, melon, and watermelon *.

Species	Number of synteny blocks	Genome coverage (%)	Frequency of synteny block size			
			<100kb	100k-1Mb	1-10Mb	>10Mb
cucumber (*C.sativus*)	463	93	71	306	86	0
melon (*C.melo*)	414	80	55	209	150	0
watermelon (*C.lannatus*)	456	86	80	219	154	3

* The scaffolds of bitter gourd (OHB3-1) genome were mapped to whole genome sequences of Cucurbitaceae plants by using Symap4.2.
