# Supplementary material for: Draft genome sequence of bitter gourd (Momordica charantia), a vegetable and medicinal plant in tropical and subtropical regions
Source: DNA Res. 2016 Dec 17;24(1):51–8. doi: 10.1093/dnares/dsw047 (PMC5381343; doi:10.1093/dnares/dsw047)
Supplement: Supplementary Data [file dsw047_Supp.zip › Suppl Tab S13.pdf]

Supplementary Table S13. Number of predicted genes encoding trypsin-inhibitor and ribosome inactivating protein in cucurbits.

		Number of genes*			
		bitter gourd	watermelon	melon	cucumber
trypsin inhibitor		34	19	24	23
Ribosome	(A-chain)	18	6	1	2
inactivating protein	(B-chain)	8	4	4	5

* According to annotation by BLAST and InterProscan analysis, number of predicted genes encoding trypsin-inhibitor or ribosome inactivating protein was count in scaffold or genome sequences.
