# Supplementary material for: Draft genome sequence of bitter gourd (Momordica charantia), a vegetable and medicinal plant in tropical and subtropical regions
Source: DNA Res. 2016 Dec 17;24(1):51–8. doi: 10.1093/dnares/dsw047 (PMC5381343; doi:10.1093/dnares/dsw047)
Supplement: Supplementary Data [file dsw047_Supp.zip › Suppl Tab S16.pdf]

Supplementary Table S16. Reference sequences (OHB3-1) mapping results of RAD-seq tags of OHB95-1A and OHB61-5*

	analyzed tags	uniquely mapped tags**	number of tag-mapped scaffolds	putative heterozygous loci***	polymorphic loci between reference sequences and inbred lines****
OHB95-1A	146,952	133,903	808	1,279	4,014
OHB61-5	115,873	104,144	791	884	3,164

* RAD-seq tag sequences were mapped to assembled draft genome sequences of bitter gourd OHB3-1 by BWA.

** Uniquely genome mapped tags were defined by mapping quality score = 37.

*** Heterozygous locus was defined, when two independent tags from the same inbred line (OHB61-5 or OHB91-5A) were mapped at the identical position of reference sequences.

**** Tag –mapped positions were defined as polymorphic loci, showing any sequence differences between reference sequences and uniquely mapped tags from OHB95-1A or OHB61-5.
