# Supplementary material for: Draft genome sequence of bitter gourd (Momordica charantia), a vegetable and medicinal plant in tropical and subtropical regions
Source: DNA Res. 2016 Dec 17;24(1):51–8. doi: 10.1093/dnares/dsw047 (PMC5381343; doi:10.1093/dnares/dsw047)
Supplement: Supplementary Data [file Suppl Fig S5.pdf › Suppl Fig S5.pdf]

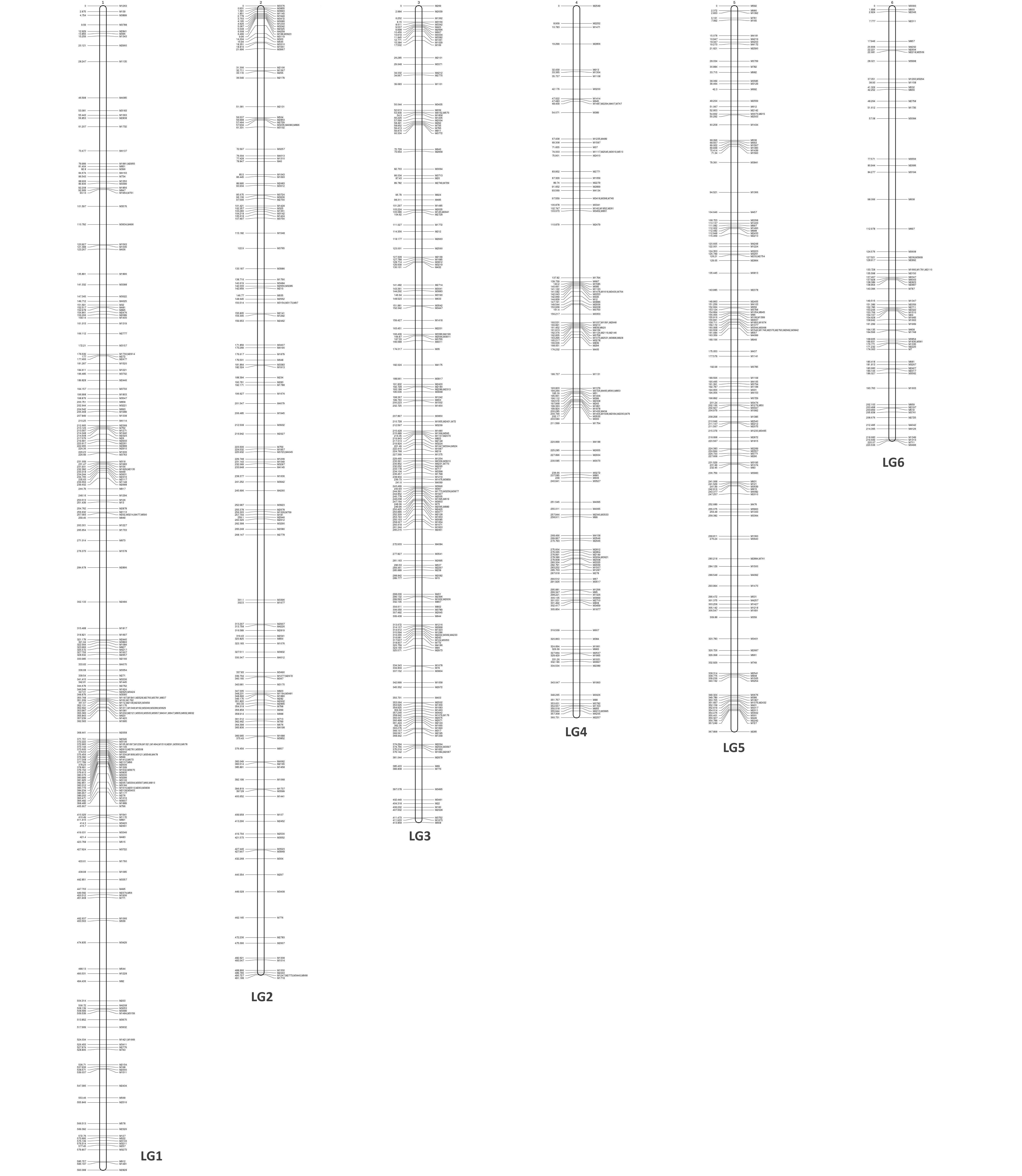

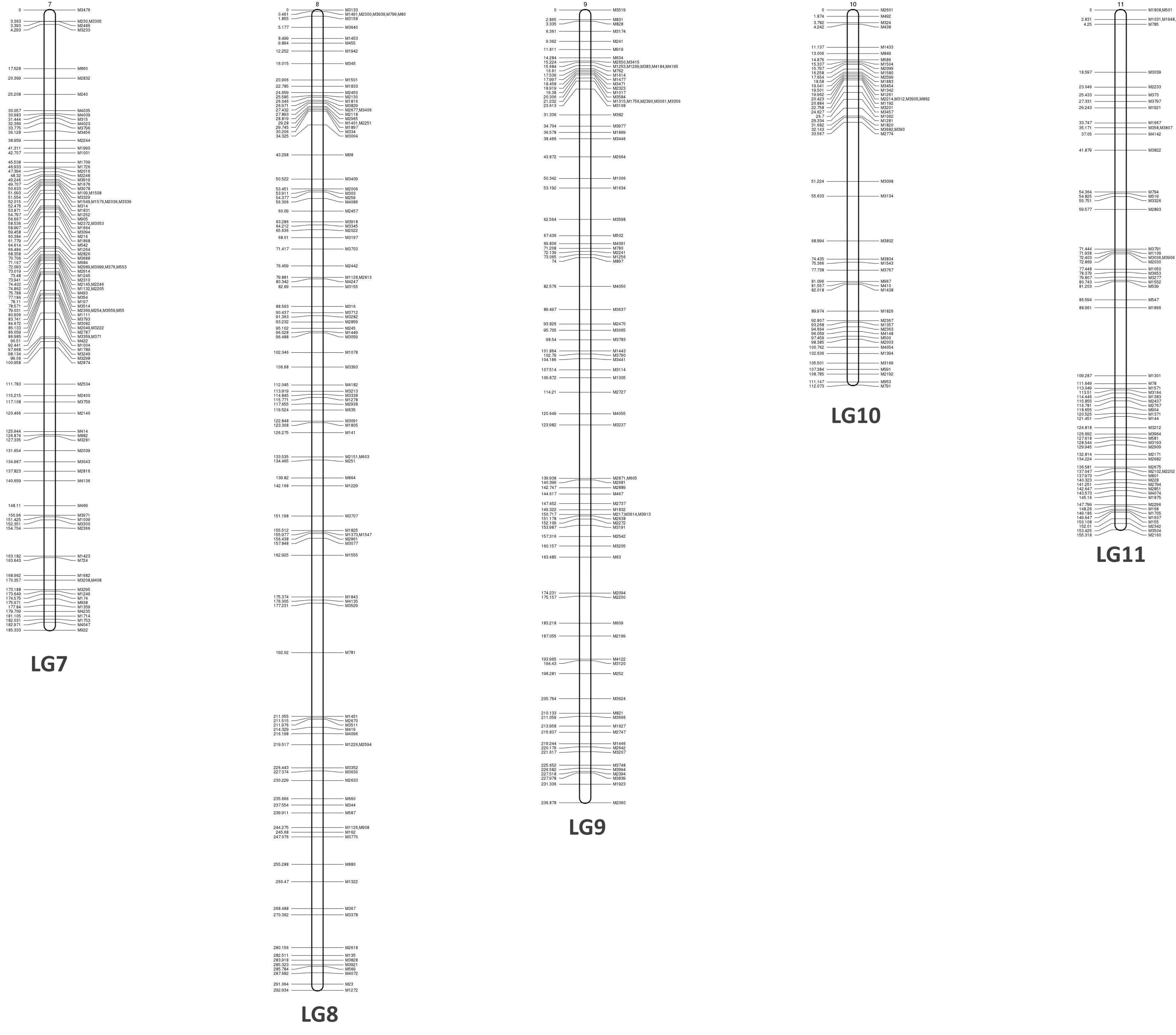

Supplementary Fig. S5. Linkage map of bitter gourd

Through RAD-seq analysis of parental lines (OHB61-5 and OHB95-1A) and their 97 F2 individuals, genotypes of 1,507 co-dominant markers were determined. Linkage map was developed by analyzing the genotypic data by using JoinMap4.1. Markers and their genetic distances (cM) In each linkage group (LG1-LG11), markers (right side) and their positions (cM, left side) are indicated. List of these markers in the map was also shown in Supplementary Table S18.
